# A Novel CCK Receptor GPR173 Mediates Potentiation of GABAergic Inhibition

**DOI:** 10.1523/JNEUROSCI.2035-22.2023

**Published:** 2023-03-29

**Authors:** Ling He, Heng Shi, Ge Zhang, Yujie Peng, Avirup Ghosh, Mengfan Zhang, Xiaofeng Hu, Chunhua Liu, Yue Shao, Shujie Wang, Lijiang Chen, Wenjian Sun, Junfeng Su, Xi Chen, Liang Zhang, Ying-Shing Chan, Duanqing Pei, Micky Tortorella, Yiping Guo, Hong Yan, Jufang He

**Affiliations:** ^1^Departments of Neuroscience; ^2^Biomedical Science; ^3^Electrical Engineering, City University of Hong Kong, Kowloon, Hong Kong; ^4^Research Institute of City University of Hong Kong, Shenzhen 518057, China; ^5^Innovation Centre for Advanced Interdisciplinary Medicine, The Fifth Affiliated Hospital of Guangzhou Medical University, Guangzhou 510799, China; ^6^Guangdong Provincial Key Laboratory of Stem Cell and Regenerative Medicine, Guangzhou Institutes of Biomedicine and Health, Chinese Academy of Sciences, Guangzhou, Guangdong 510070, China; ^7^School of Biomedical Science, University of Hong Kong, Hong Kong; ^8^Center of Regenerative Medicine and Health, Hong Kong Institute of Science and Innovation, Hong Kong Academy of Sciences, Hong Kong

**Keywords:** CCK receptor, CCK-GABA, enhancement of the inhibition, GPR173, inhibition, inhibitory plasticity

## Abstract

Cholecystokinin (CCK) enables excitatory circuit long-term potentiation (LTP). Here, we investigated its involvement in the enhancement of inhibitory synapses. Activation of GABA neurons suppressed neuronal responses in the neocortex to a forthcoming auditory stimulus in mice of both sexes. High-frequency laser stimulation (HFLS) of GABAergic neurons potentiated this suppression. HFLS of CCK interneurons could induce the LTP of their inhibition toward pyramidal neurons. This potentiation was abolished in CCK knock-out mice but intact in mice with both CCK1R and 2R knockout of both sexes. Next, we combined bioinformatics analysis, multiple unbiased cell-based assays, and histology examinations to identify a novel CCK receptor, GPR173. We propose GPR173 as CCK3R, which mediates the relationship between cortical CCK interneuron signaling and inhibitory LTP in the mice of either sex. Thus, GPR173 might represent a promising therapeutic target for brain disorders related to excitation and inhibition imbalance in the cortex.

**SIGNIFICANCE STATEMENT** CCK, the most abundant and widely distributed neuropeptide in the CNS, colocalizes with many neurotransmitters and modulators. GABA is one of the important inhibitory neurotransmitters, and much evidence shows that CCK may be involved in modulating GABA signaling in many brain areas. However, the role of CCK-GABA neurons in the cortical microcircuits is still unclear. We identified a novel CCK receptor, GPR173, localized in the CCK-GABA synapses and mediated the enhancement of the GABA inhibition effect, which might represent a promising therapeutic target for brain disorders related to excitation and inhibition imbalance in the cortex.

## Introduction

Cholecystokinin (CCK), the most abundant and widely distributed neuropeptide in the CNS (Crawley and Corwin, 1994), colocalizes with glutamate (Morino et al., 1994), GABA (Somogyi et al., 1984; Markram et al., 2004), vasoactive intestinal peptide (VIP; Kosaka et al., 1985), dopamine (Hökfelt et al., 1980), and serotonin (Van Der Kooy et al., 1981). Repetitive firing or stimulation of GABAergic neurons, especially at high frequency, induces neuroplasticity of inhibition and long-term potentiation of inhibitory synapses (iLTP) in the neocortex and hippocampus (Komatsu, 1994; Caillard et al., 1999; Nugent et al., 2007). CaMKII-dependent phosphorylation of GABA_A_ receptor (GABA_A_R)-induced postsynaptic iLTP has been recognized (Petrini et al., 2014; He et al., 2015; Chiu et al., 2018). In addition, cortical interneuron-mediated iLTP is expressed in other forms, including NMDA receptor-dependent spike-timing-dependent plasticity in the auditory cortex (AC; Vickers et al., 2018), brain-derived neurotrophic factor receptor-TrkB signaling-dependent iLTP in the developing AC (Xu et al., 2010), and GABA_B_R-dependent forms of iLTP in the visual cortex (Komatsu, 1996). In the hippocampal network, synapses from parvalbumin (PV)-expressing interneurons (INs; PV-INs) or somatostatin (SOM)-expressing interneurons (SOM-INs) can undergo two unique forms of plasticity relying on T-type voltage-gated Ca^2+^ channel (VGCC) activation, long-term depression (LTD; PV-iLTD) or SOM-iLTP, respectively (Udakis et al., 2020). These forms of iLTP indicate that different mechanisms are involved depending on brain areas, neuronal connections, and brain activities.

CCK-related inhibitory plasticity has been characterized by the aspects of brain regions, cell types, and synapses. CCK-mediated inhibitory plasticity requires high-frequency stimulation (HFS) as well as CCK2R activation and astrocytic ATP release in the dorsomedial hypothalamus via increased GABA release (Crosby et al., 2015, 2018). CCK increases the frequency of spontaneous ISPCs (sIPSPs) and currents (sIPSCs) in the basolateral amygdala (Chung and Moore, 2007). Recently, Martinez Damonte et al. (2022) reported that HFS-induced somatodendritic-released CCK potentiates GABAergic synapses onto ventral tegmental area dopamine cells. Also, Dudok et al. (2021) found that CCK basket cell activity inversely scales with pyramidal cell ensemble activity during locomotion and rest. However, cortical CCK-IN-induced iLTP is not fully elucidated at the presynaptic or postsynaptic levels. Despite CCK being a marker for cortical GABAergic neurons (Kubota and Kawaguchi, 1997), its functional role in cortical GABA inhibitory synapses is still unknown, as well as which CCK receptor is involved in this process.

CCK1R and CCK2R, two known CCK receptors in the mammalian brain (Moran et al., 1986; Wank et al., 1992; Noble and Roques, 1999), are G-protein-coupled receptors (GPCRs). CCK1R links hippocampal LTP and spatial memory (Harro and Oreland, 1993). CCK2R mediates LTP in excitatory synapses and enables associative memory encoding between two sounds, sound and light, or sound and fear (Li et al., 2014; Chen et al., 2019; Zhang et al., 2020; Feng et al., 2021). Given that CCK mediates excitatory and inhibitory circuit LTP in different brain regions, the following question arises: Does CCK released from GABAergic neurons mediate iLTP in the cortex via known CCK receptors?

We performed *in vivo* extracellular and *in vitro* patch-clamp recording experiments to address the above question in the present study. We found that iLTP was not induced in CCK-KO mice but in CCK1R/2R double KO mice, implying that a novel CCK receptor mediates iLTP. Our next objective was to identify this novel CCK receptor. We then screened the homology and similarities of known GPCRs to CCK1R and CCK2R using a bioinformatics algorithm to shortlist candidate receptors, followed by multiple cell-based assays to validate their binding properties with the CCK ligand. We also examined the colocalization of candidate GPCRs and CCK-GABAergic synapses and their distribution in excitatory and inhibitory neurons. Finally, we performed loss-of-function studies in cell and brain slice assays. In this manner, we identified GPR173 as a novel CCK receptor involved in the iLTP of CCK-INs in the cortex.

## Materials and Methods

### Animals

All procedures were in accordance with guidelines approved by the Animal Subjects Ethics Sub-Committee of the City University of Hong Kong. The following transgenic mice were used: Vgat-ires-Cre (^Slc32a1tm2(Cre)Lowl^/J, Vgat-Cre, C57 background, The Jackson Laboratory), mCCK-cKO (CCK-cKO, C57BL/6J background, Cyagen), Vgat-Cre-CCK-cKO (cross of Vgat-Cre and CCK-cKO mice to specifically knock out CCK in GABAergic neurons), CCK-ires-Cre (Cck^tm1.1(Cre)Zjh^/J, C57BL/6J background; for short CCK-Cre, The Jackson Laboratory), PV-ires-Cre (Pvalb^tm1(cre)Arbr^/J, PV-Cre, C57BL/6J background, The Jackson Laboratory), CCK-CreER (Cck^tm2.1(Cre/ERT2)Zjh^/J, CCK-KO, C57BL/6J background, The Jackson Laboratory), CCK-AR/BR-KO (Cckar^tm1Kpn^Cckbr^tm1Kpn^/J, CCK1R/2R-KO, 129S1 background, The Jackson Laboratory), and C57BL/6J mice. We used standard procedures and recommended primers for genotyping. The primer pairs used for each mouse line are listed in Table 1.

**Table 1.** Reagents, cell lines, mice and oligonucleotides

Reagent resource	Source	Identifier (catalog #)
Anti-GAD67	Millipore	Mab5406
Anti-ChR2	ARP	03–651180
Anti-CCK	Sigma	C2581
Anti-PV	Abcam	Ab11427
Anti-GABA_A_R	Santa Cruz	Sc-376282
Anti-GABA_A_R	Abcam	Ab7291
Anti-mCherry	Thermo Fisher Scientific	M11217
Anti-GFAP	Santa Cruz Biotechnology	Sc-33673
Anti-Camkll	Abcam	Ab5683
Anti-Camkll	Abcam	Ab325403
Anti-Flag	Cell Signaling Technology	CST-8146
Anti-HA	Cell Signaling Technology	CST-3724
Anti-Synaptophysin 1	Synaptic Systems	101 004
Anti-GPR173	Thermo Fisher Scientific	PA5-50976
Anti-GPR85	Thermo Fisher Scientific	PA5-33776
Anti-CCK1R	Novus	AF2680
Anti-CCK2R	Affinity	DF4861
Anti-GPR15	Affinity	DF4965
Anti-BRS3	Santa Cruz Biotechnology	sc-271712
Anti-GPR17	Santa Cruz Biotechnology	sc-514723
Anti-GPR135	Affinity	DF4948
Anti-GPR132	Santa Cruz Biotechnology	sc-137112
Anti-TAAR9	Novus	NLS1969
Anti-GPR19	Affinity	DF2729
Anti-GPR34	Affinity	DF4972
Anti-GPR83	Affinity	DF2768
Anti-GPR45	Absin	abs112926
Anti-TAAR5	Novus	NBP1-68902
Anti-GPR20	Affinity	DF2730
Goat anti-Gp 405	Abcam	ab175678
Goat anti-Gp 488	Abcam	ab150185
Goat anti-mouse 488	Abcam	ab150113
Goat anti-rabbit 647	Abcam	ab150079
Goat anti-mouse 568	Abcam	ab175473
Goat anti-rabbit 488	Abcam	ab150077
Goat anti-mouse 594	Thermo Fisher Scientific	35511
Goat anti-rat 488	Thermo Fisher Scientific	A11006
Donkey anti-mouse 488	Jackson ImmunoResearch	715–545-150
Donkey anti-mouse 594	Jackson ImmunoResearch	715–585-150
Donkey anti-mouse 647	Jackson ImmunoResearch	715–605-150
Donkey anti-rabbit 488	Jackson ImmunoResearch	711–545-152
Donkey anti-rabbit 594	Jackson ImmunoResearch	711–585-152
Goat anti-rabbit 750	Thermo Fisher Scientific	A-21039
Goat anti-mouse 750	Thermo Fisher Scientific	A-21037
Goat anti-rat 647	Abcam	ab150159
Donkey anti-rabbit 647	Jackson ImmunoResearch	711–605-152
A-71623	Tocris Bioscience	2411
CCK4	Bankpeptide	N/A
CCK8s	Bankpeptide	N/A
PNX	Bankpeptide	N/A
CCK8	Bankpeptide	N/A
HA-(ε-Ahx)_2_	Bankpeptide	N/A
HA-(ε-Ahx)_2_-CCK4	Bankpeptide	N/A
HA-(ε-Ahx)_2_-CCK8ns	Bankpeptide	N/A
HA-(ε-Ahx)_2_-CCK8s	Shanghai Ke Biochem	N/A
Calcium imaging assay kit	AAT Bioquest	36315
CHO-CCK1R (CHO cell line stably expressing CCK1R)	This article	N/A
CHO-CCK2R (CHO cell line stably expressing CCK2R)	This article	N/A
CHO-GPR173 (CHO cell line stably expressing GPR173)	This article	N/A
CHO	Cell Bank of Guangzhou Institutes of Biomedicine and Health, Chinese Academy of Sciences	N/A
HEK293T	ATCC	CRL-3216
Vgat-Cre: Vgat-ires-Cre^S^*^lc^*^32^*^a^*^1^*^tm^*^2(C^*^re^*^)L^*^owl^*/J	The Jackson Laboratory	JAX: 016962
CCK-cKO: CCK conditional KO	Cyagen	N/A
CCK-Cre: CCK-ires-Cre (Cck*^tm^*^1.1(C^*^re^*^)Z^*^jh^*/J)	The Jackson Laboratory	JAX: 019021
PV-Cre: PV-ires-Cre (Pvalb*^tm^*^1(^*^cre^*^)A^*^rbr^*/J)	The Jackson Laboratory	JAX: 017320
CCK1R/2R-KO: CCK-AR/BR-KO (Cckar*^tm^*^1K^*^pn^* Cckbr*^tm^*^1K^*^pn^* /J)	The Jackson Laboratory	JAX: 006365
CCK-KO: CCK-CreER (Cck*^tm^*^2.1(C^*^re^*^/ERT2)Z^*^jh^*/J)	The Jackson Laboratory	JAX: 012710
Primer for Vgat-Cre	BGI	CAGGGCGATGTGGAATAGAAA CTTCGTCATCGGCGGCATCTG CCA AAAGACGGCAATATGGT
Primer for CCK-cKO	BGI	GAAGGATGCCAGGAAAGGTGGTAGC CACATGGAAACACAGCCTATCGTTCC
Primer for CCK-Cre	BGI	GAGGGGTCGTATGTGTGGTT GGGAGGCAGATAGGATCACA TGGTTTGTCCAAACTCATCAA

### Generation of Vgat-Cre-CCK-cKO mice

We crossed homozygous male Vgat-Cre (+/+) mice with homozygous female CCK-cKO (+/+) mice to establish first-generation hemizygous Vgat-Cre (+/−)-CCK-cKO (+/−) mice. We obtained Vgat-Cre (+/+)-CCK-cKO (+/+) after mixing male and female hemizygous Vgat-Cre (+/−)-CCK-cKO (+/−) mice.

### Local virus injection in the AC

We injected mice (6–8 weeks old) with atropine (0.05 mg/kg, i.m.; Sigma-Aldrich) to inhibit tracheal secretions before the anesthetics (pentobarbital sodium, 80 mg/kg, Dorminal 20%, Alfasan Woerden-Holland) were injected intraperitoneally. Body temperature was maintained between 37 and 38°C with a heating pad (RWD Life Science). We also applied a local anesthetic (xylocaine, 2%) to the incision site for analgesia. We performed craniotomy with two holes of 300 µm diameter on head-fixed mice to access the AC (−2.0 to −3.0 mm posterior to the bregma and −4.0 to −4.3 mm to the midline) using a stereotactic device (RWD Life Science). We injected a double loxP-flanked DIO (also known as Double-Floxed Inverted Open reading frame) Cre-dependent AAV vector expressing channelrhodopsin-2 variants (ChETA) fused with an enhanced yellow fluorescent protein (eYFP) AAV-EF1a-DIO-ChETA-eYFP, 8E + 12 vector genome (vg)/ml) or its control (AAV-EF1α-DIO-eYFP, 5E + 12 vg/ml; University of North Carolina Vector Core) was injected into the AC (300 nl of AAV vector at 30 nl/min at four sites using a Nanoliter Injector (World Precision Instruments) of Vgat-Cre and Vgat-Cre-CCK-cKO mice.

For brain slice recording experiments, we injected rAAV9-mDlx-DIO-ChR2-mCherry-WPRE-pA (5.18E + 12 vg/ml; BrainVTA) into the AC of CCK-Cre and PV-Cre mice.

For experiments in CCK1R/2R-KO and CCK-KO mice, we injected a mixture of AAV9-mDlx-Cre-WPRE-pA (1.42E + 12 vg/ml; BrainVTA) and rAAV9-mDlx-DIO-ChR2-mCherry-WPRE-pA (2.09E + 12 vg/ml) into the AC of mice.

### Optogenetics: *in vivo* local field potential and unit recordings

#### *In vivo* recordings

We performed *in vivo* recordings 3–4 weeks after virus injection, as previously described (Li et al., 2014; Chen et al., 2019; Zhang et al., 2020). We injected mice with atropine to inhibit tracheal secretions 15 min before urethane sodium anesthetic injection (2 × g/kg, i.p.; Sigma-Aldrich. We performed a craniotomy to access the AC (−1.8 to −3.5 mm posterior to bregma and −3.5 to −4.5 mm to the midline).

We inserted a recording borosilicate glass (World Precision Instruments) electrode array (pulled with a Sutter-87 puller) 300–500 µm deep. We placed a laser fiber (200-µm diameter, RWD Life Science) on the surface of the AC where the virus was infused. We controlled a stepping-motor microdriver outside the soundproofed chamber to advance the electrode array. Signals were preamplified, filtered with a bandwidth of 1–5000 Hz, and stored in a Tucker-Davis Technologies (TDT) workstation (OpenEx). The TDT workstation generated a noise burst of 100 ms duration that led to a coupled electrostatic speaker as the auditory stimulus [AS; 50–105 dB, sound pressure level (SPL)]. When the AS reached the animal, a condenser microphone was used to measure and adjust the sound intensity of the speaker (Center Technology). A laser generator (Newdoon) delivered a laser pulse (5 ms duration) at a 473 nm wavelength. We set the intensity of the laser at ∼0–10 mW at the end of the laser fiber.

We measured the input-output curve between the sound intensity (60–95 dB, SPL) and the amplitude of the local field potential (LFP). We adopted the intensity that evoked 50% of the maximum response as the sound intensity. We also measured the input-output curve between the laser power (0–15 mW) and the amplitude of the LFP to help select the laser power. We adopted the laser power that evoked 50–70% of the maximum response as the stimulation power in every experiment.

We confirmed viral expression by examining the laser-induced suppressive effect on spontaneous neuronal responses. We selected the interval between the laser and AS to achieve the optimal suppressive effect of laser-activated inhibitory neurons on neuronal responses to the forthcoming AS. We set a gap of 50 ms for subsequent experiments. The LFP traces were calculated with MATLAB and plotted over time.

We examined spontaneous neuronal activities and responses to the laser and AS combination as units and LFP (0.1 Hz) for 15 min. We delivered a single-pulse laser (3–5 mW, 5 ms duration) in 0.1 Hz repetitions 50 ms before the AS. We recorded for 15 min before (baseline) and 60 min after high-frequency laser stimulation (HFLS; 5 pulses at 40 Hz, repeated for 40 trials at 0.1 Hz). We examined the frequency of HFLS as a parameter to confirm laser-evoked responses, which helped us select the laser frequency as our desired HFLS and remove light/heat effects (1–120 Hz, repeated for 20 trials at 0.1 Hz).

#### *In vivo* LFP analysis

We averaged LFP amplitudes across every six trials (MATLAB), representing a 1 min recording, and plotted the representative waveforms with MATLAB.

We present group data as the mean ± SEM. We used 3–11 mice per condition for each experiment. We randomly selected male or female mice for each experiment. For all *in vivo* electrophysiology experiments, *n* refers to the number of recording sites per condition, and *N* refers to the number of animals. We analyzed data in a blinded manner. We performed a two-way ANOVA and Tukey's *post hoc* tests for experiments involving two independent variables using IBM SPSS Statistics software or Microsoft Excel, assuming a normal distribution when *n* was too small to determine distribution. We used OriginPro 8.5 software to show group data in all figures.

#### Unit waveform processing

We used commercial hardware and software (OpenEx) to analyze the waveform from every unit with OpenBridge, Plexon Offline Sorter, and NeuroExplorer. We recorded multiunits primarily with the current electrode preparation.

We discriminated units from the PLX file using principal component analysis with the Plexon Offline Sorter. We adopted two criteria to ensure the quality of recorded units, (1) a signal larger than three times the noise band and (2) no more than 1% of the interspike intervals shorter than 2 ms. We considered only those neurons with at least 1000 detected spikes within 15 min in our analysis.

We opened the sorting in the NEX5 file by NeuroExplorer. We plotted rasters and histograms for units using perievent rasters with specific, fixed reference events (laser, AS, or sweep) with a 5 ms bin width. We extracted the single and averaged waveforms into OriginPro 8.5. Counts per bin (5 ms) depending on the duration of the fixed reference event time for a comparable experiment were used for firing rate quantification. We calculated the average firing rate during the 100 ms AS presentation. We excluded the period between the laser and 100 ms after the AS presentation from the spontaneous firing rate quantification because such firing corresponded to the indirect activation of GABAergic neurons or a delayed noise response.

### *In vitro* patch-clamp recordings

#### Mouse brain slice preparation

In the *in vivo* brain slice recording experiments, we adopted a protective cutting and recovery method to prepare acute brain slices from CCK-Cre, PV-Cre, CCK-KO, and CCK1R/2R-KO mice to achieve a higher success rate for patch clamping, as previously described (Chen et al., 2019). Briefly, we perfused the anesthetized mice transcardially with *N*-methyl-d-glucamine/artificial CSF (NMDG-aCSF) containing the following (in mm): 92 NMDG, 2.5 KCl, 1.25 NaH_2_PO_4_, 30 NaHCO_3_, 20 HEPES, 25 glucose, 2 thiourea, 5 Na-ascorbate, 3 Na-pyruvate, 0.5 CaCl_2_·4H_2_O, and 10 MgSO_4_·7H_2_O, pH 7.3–7.4) then extracted and cut the brain into 300-µm-thick sections. We transferred the slices to NMDG-aCSF for 10 min at 32–34°C to allow protective recovery and then to room temperature HEPES-aCSF containing the following (in mm): (92 NaCl, 2.5 KCl, 1.25 NaH_2_PO_4_, 30 NaHCO_3_, 20 HEPES, 25 glucose, 2 thiourea, 5 Na-ascorbate, 3 Na-pyruvate, 2 CaCl_2_·4H_2_O, and 2 MgSO_4_·7H_2_O, pH 7.3–7.4) for at least 1 h before recording.

#### Whole-cell recordings

We performed whole-cell recordings with a MultiClamp 700B amplifier and Digital 1440A digitizer (Molecular Devices) targeting the AC in the brain slices in room temperature aCSF containing the following (in mm): 119 NaCl, 2.5 KCl, 1.25 NaH_2_PO_4_, 24 NaHCO_3_, 12.5 glucose, 2 CaCl_2_·4H_2_O, and 2 MgSO_4_·7H_2_O, ∼25°C). We pulled patch pipettes with resistance between 3 and 5 MΩ from borosilicate glass (World Precision Instruments) on a Sutter-87 puller. The intracellular solution contained the following (in mm): 145 K-Gluconate, 10 HEPES, 1 EGTA, 2 Mg-ATP, 0.3 Na_2_-GTP, and 2 MgCl_2_, pH 7.3, 290–300 mOsm. We made recordings after giga-ohm seal formation and terminated if *R*s changed >20%. We selected pyramidal neurons (PC) based on their pyramidal-like shape and their regular spiking firing pattern by injecting step currents. We infused Biotin Alexa Fluor 488 after recording to confirm the patched pyramidal neuron. We applied laser stimulation through an optical fiber placed ∼200 µm from the recording neuron using Aurora-220 (473 nm, Newdoon) to activate ChR2^+^ neurons (GABAergic). We selected neurons that responded to the laser pulse only in our experiment. We delivered HFLS (10-pulse burst at 40 Hz, repeated for 10 trials) or low-frequency laser stimulation (LFLS; 1 Hz, 100 pulses) with a 15 s interval. We recorded IPSCs in response to the laser stimulation (1 pulse per 15 s, 5 ms in duration) under voltage-clamp recording mode (holding at −60 mV to −50 mV) for 5 min as the baseline before and for 25 min after HFLS or LFLS. In a subset of experiments, we applied 200 nm sulphated CCK8 (CCK8s) via bath application 3 min before LFLS. In another subset of experiments, we applied devazepide in the recording chamber via bath application (60 nm) 3 min before HFLS. Drugs were naturally washed out. For the normalization of recorded IPSCs in the CCK-Cre, PV-Cre, CCK-KO, and CCK1R/2R-KO mice, we selected only patched neurons with IPSCs >35 pA.

### Searching for potential candidates of CCK receptors by bioinformatics methods

#### Proteomics screening in the GPCR pool in comparison with CCK1R and CCK2R

We downloaded ∼200 GPCRs, both orphans and characterized receptors, known from the literature as FASTA formatted sequences obtained from UniProt (https://www.uniprot.org). All FASTA formatted sequences were merged into one file for further analysis. For the hierarchical cluster and scores for CCK1R/2R, we set two parameters as follows: (1) We selected binding sites from the GPCRs secondary structure (extramembrane) based on the CCK binding domains of CCK1R and CCK2R, and (2) we compared amino acid sequences based on the hamming distance. After identifying all proteins with these two parameters, we selected GPCRs with high similarity scores for both CCK1R and CCK2R. We analyzed the data with the online free software NetSurfP version 1.1 (new version available at https://services.healthtech.dtu.dk/service.php?NetSurfP-1.1). NetSurfP predicts amino acid surface accessibility and secondary structure in an amino acid sequence. This study validated total similarity scores for CCK1R and CCK2R above 30 without additional manual inspection.

#### Percentage identity of CCK1R, CCK2R, and 79 orphan GPCRs

We examined the percentage identity matrix for 79 orphan GPCRs with CCK1R and CCK2R. Percentage identity is the quantitative correlation estimation between two sets of sequences. Closely correlated species have a higher percentage identity for a given amino acid sequence. Hence, percentage identity reflects relatedness to some extent. The sequences of 79 orphan GPCRs, together with CCK1R and CCK2R proteins, were analyzed and plotted by a custom-designed program.

#### Peptide synthesis

Peptides and modified peptides were chemically synthesized by Bankpeptide Biotechnology, Synpeptide, or Shanghai Ke Biochem. HPLC analysis results ensured that the purity of peptides was >97%. We list peptide and modified peptide sequences in Table 1.

#### Plasmid construction

For CHO-GPCR cell line construction, human coding sequences (CDSs) of the GPCRs were cloned into multiple cloning sites of pLVX-puro. For the cell surface binding assay, Flag (at the N terminus of GPCRs)-tagged human GPCR coding sequences were cloned into pCDNA3.1(+) plasmids. For the β-arrestin2 recruitment assay, our Tango-GPCR plasmids were constructed based on pCDNA3.1(+) as described previously (Kroeze et al., 2015), except that we cloned the full CDS of human GPCRs into pCDNA3.1(+). For the β-arrestin1/2 recruitment assay, the β-arrestin1-linker-TEV (tobacco etch virus) protease-HA tag was cloned into pCDNA3.1(+), in which β-arrestin1 was the human CDS (NM_004041.5), TEV protease was the 2040-2279-aa fragment of TEV Nla protease (accession #M15239.1, GenBank), and linker sequence was the (Gly_4_Ser)_2_ coding sequence. All final recombinant vectors were confirmed by sequencing analysis.

#### Cell surface binding assay

We transfected HEK293T cells with pCDNA3.1(+)-Flag-GPCR (purchased from Public Protein/Plasmid Library or made in our laboratory based on a pCDNA3.1(+) backbone). We incubated the cells with HA-(ε-Ahx)_2_ (as a negative control) or HA-(ε-Ahx)_2_-CCK8s dissolved in HHBS solution (including 20 mm HEPES, pH 7.4) for 30 min at room temperature 48 h after transfection by Lipo6000 (Beyotime Biotechnology). After three washes with HHBS, cells were fixed in 4% paraformaldehyde (PFA) and 4% sucrose in PBS for 15 min at room temperature. Cells were blocked in 5% normal goat serum in PBS, and staining was performed in detergent-free conditions without cell permeabilization after three washes by PBS. We used primary antibody rabbit anti-HA (catalog #8146, Cell Signaling Technology), anti-GPR173 (catalog #PA5-50 976, Thermo Fisher Scientific), and mouse anti-Flag (catalog #3724, Cell Signaling Technology) to detect HA and Flag tags. Images were captured on a Leica SP8 confocal microscope. Images within each experiment were collected using identical laser power, offset, and gain.

#### Immunohistochemical staining

For immunohistochemistry, we deeply anesthetized mice with an overdose of pentobarbital sodium. We transcardially perfused mice with 30 ml cold PBS and 30 ml 4% (w/v) PFA. Brain tissue was removed, postfixed with 4% PFA, and treated with 30% (w/v) sucrose in 4% PFA at 4°C for 2–3 d. Brain tissue was sectioned on a cryostat (30 µm for standard staining and 10 µm for super-resolution imaging; Leica CM3500) and preserved with antifreeze buffer (20% v/v glycerin and 30% v/v ethylene glycol diluted in PBS) at −20°C. For immunostaining and statistical analysis of colabeled neurons and axonal synapses in the AC, serial sections containing the AC were selected for analysis. Brain sections were rinsed three times with PBS and blocked with blocking buffer (10% v/v goat serum in PBS with 0.2% v/v Triton X-100) for 2 h at room temperature. Sections were incubated with primary antibodies (Table 1) at 4°C for 24–36 h. After washing four times in PBS (each time for 10 min), sections were incubated with the corresponding fluorophore-conjugated secondary antibodies (Table 1) for 2.5–3 h at 25°C. Sections were then rinsed with PBS three times before DAPI staining (1:5000 v/v diluted in PBS) or mounting. We mounted all sections with 70% v/v glycerin in PBS (standard staining) or imaging buffer (super-resolution imaging) on slides.

#### Image acquisition and analysis

Image acquisition (10×, 20×, 63×, and 100× magnification) was performed using a Nikon A1hD25 confocal microscope. The confocal microscope was equipped with a time delay integration camera and performed line scanning with fast acquisition at a high resolution of the fluorescent signal.

We took images at 20× magnification and montaged them with the same laser settings between parallel studies. We optimized the gain and exposure parameters for each image. We captured Z-stacks throughout the tissue for magnified images showing labeling details. For quantification of colocalization, neurons expressing the indicated reporter were counted using only the corresponding color channel. Then, among these cells, the number of neurons coexpressing the marker of interest was counted by Nikon imaging analysis software (NIS-Elements Viewer 5.21). Bright spot detection was performed in the fields of interest in brain regions with the software under an appropriate fluorescent threshold. A neuron was positive for a given marker if the corresponding signal was above the background fluorescence. The ratio of neurons coexpressing both markers over the total number of cells expressing only the reporter was then calculated.

The *n* in the figures indicates the number of representative biological replicates, and *N* indicates the number of independent animals. At least three biological replicates per data point were included for all quantifications used in the present study.

#### Super-resolution imaging and processing

The staining process for super-resolution imaging was the same as immunohistochemical staining except for differences in secondary antibody and mounting. Two fluorophores were used (Alexa Fluor 647 in combination with Alexa Fluor 750) as secondary antibodies. To immobilize a labeled slice, we first applied 200 μl fluorescent beads (Thermo Fisher Scientific) onto the 1.5-μm-thick cover glass with 120°C heating to attach tightly. After several washes with double-distilled H_2_O, a well-labeled slice of the AC was gently attached to the cover glass where beads were attached. After drying, the slice was sealed on the slide with imaging buffer (150 mm Tris, pH 8, 1% glycerol, 1% glucose, 10 mm NaCl, 1% β-Mercaptoethanol, 0.5 mg/ml glucose oxidase, 40 μg/ml catalase). We performed dSTORM imaging in a freshly prepared imaging buffer to slow the speed of fluorophores quenching during imaging acquisition. STORM imaging was performed on a 20× and 100× Olympus microscope [NanoBioImaging SRiS 2.0 (STORM) Super-Resolution Microscope, Hong Kong University, Olympus] and analyzed with ImageJ software (National Institutes of Health).

In super-resolution capture mode, we first locked slice focus with Narrow Band Imaging Rohdea 3.0 under 20× magnification by capturing the signal of fluorescent beads attached to slides. A Z-stack of fluorescent beads at this moment acted as a reference. We first turned on a 405 nm laser to activate the two fluorophores. Under illumination at 10–15% of the maximum output power of 656 nm and 750 nm lasers, we observed a preview of the target signal before imaging. We acquired 3000 frames with 656 nm and 750 nm laser illumination at 30–50% maximum output power to activate the two fluorophores. We estimated drift using the inbuilt function in SRiS 2.0 and applied correction during analysis. The raw data, single-channel Excel file, and raw data files were autogenerated for analysis after capturing.

The Excel file was directly imported into ImageJ. After dataset reconstruction with the same parameters for both channels, red and green RGB (red, green, and blue) colors were used to differentiate the fluorophores in the two channels. The two colors were merged into one channel, forming the overlay images.

The Pearson correlation coefficient (PCC) and Manders' overlap coefficient (MOC) are common metrics for measuring the predictability of colocalization (Aaron et al., 2018). In more mathematical terms, the PCC can be thought of as the covariance between the two images, normalized by the product of their SDs.

#### Generation of GPCR-overexpressing cell line

We maintained HEK293T cells in DMEM/high glucose medium (Thermo Fisher Scientific) supplemented with 10% FBS (Natocor Industria Biolóica), 1% non-essential amino acids (Invitrogen), and 1% Glutamax (Invitrogen) at 37°C in a humidified atmosphere of 5% CO_2_. CHO and infected CHO cells (CHO-GPCRs) were cultivated at 37°C in a humidified atmosphere with 5% CO_2_ in DMEM/F-12 medium (Thermo Fisher Scientific) supplemented with 10% FBS (Natocor Industria Biológica). To obtain lentivirus, we seeded the HEK293T cells on reaching 70–90% confluency in six-well tissue culture plates. We incubated them for 10–12 h in a mixture of calcium phosphate transfection reagent [31.25 µl 2 m CaCl_2_ solution, 250 µl 2× HBS (0.05 mol/l HEPES, 0.012 mol/l D-(+)-Glucose, 0.28 mol/l NaCl, 0.023 mol/l KCl, 0.0015 mol/l Na_2_HPO_4_, and 218.75 µl H_2_O)] and 1 ml fresh medium with 4 µg pLVX-puro-GPCR plasmid (Public Protein/Plasmid Library), 3 µg pSPAX2 plasmid (catalog #12260, Addgene), and 1.2 µg pMD2.G plasmid (catalog #12 259, Addgene). We cultured the cells for an additional 36 h in their normal medium before collecting the virus supernatant and infecting CHO cells.

Meanwhile, we seeded ∼1 × 10^6^ CHO cells in six-well plates with 0.5 ml virus supernatant per well for 12 h and then mixed and added 0.1% polybrene (Sigma-Aldrich) in 1 ml fresh normal medium. We performed the second infection for 24 h and replaced the virus supernatant mix with a fresh normal medium of CHO after 6–8 h of each infection. Two days after the second infection, we added puromycin (Invitrogen) at a final concentration of 10 µg/ml for 1 week. To obtain high-expression cell lines, we isolated single cells by limited dilution in 96-well plates and performed quantitative RT-PCR (qRT-PCR) after 1 month of culture. Finally, the highest-expressing cell line was chosen for further characterization.

#### GPR173 knockdown with shRNA (Gpr173) in CHO-GPR173 cells

The coding sequence of Hygromycin B was PCR-amplified by Phanta Max Super-Fidelity DNA Polymerase (Vazyme Biotech) from other plasmids. The purified Hygromycin B fragment and pLKO.1-puro plasmid (catalog #8453, Addgene) was digested by BamHI and KpnI restriction enzyme (New England Bio Labs). We separated the resulting fragments by 1.0% agarose gel electrophoresis and retrieved them using a DNA retrieval kit (Tiangen Biotech). We combined the two BamHI and KpnI fragments with T4 DNA ligase (New England Bio Labs). We termed the generated plasmid “pLKO.1-hygro.” We then designed and cloned the selected shRNA sequence into the pLKO.1-hygro plasmid by AgeI/EcoRI sites. We determined the most effective shRNA (*Gpr173*) sequence, ACGTGGGCACCTACAAGTTTA (with scramble sequence as follows: CCTAAGGTTAAGTCGCCCTCG). We generated a GPR173 knock-down cell line using the method described above. After lentivirus infection of CHO-GPR173 cells, we screened cells with 400 µg/ml Hygromycin B (Thermo Fisher Scientific) for at least 1 week.

#### qRT-PCR

Primer synthesis was conducted by Generay Biotechnology using the following sequences: *gpr173*-TCTGGTCACCCTACATCGTG/CAGTAGGGTTCTCTGGGAGC and *actb-*CCTCTATGCCAACACAGTGC/CCTGCTTGCTGATCCACATC.

After extracting total RNA using an RNAsimple Total RNA Kit (TIANGEN Biotech), we used 1.6 µg RNA and a FastKing RT Kit (TIANGEN Biotech) to generate cDNA according to the instructions from the manufacturer. Then, the primers were amplified with SYBR Green Mix (TIANGEN Biotech) through the 2^−ΔCt^ method. The *actb* was used as an endogenous control gene for *gpr173*. We repeated all procedures three times in triplicate.

#### Ca^2+^ imaging assay

For Ca^2+^ mobilization by a confocal microscope, ∼8 × 10^4^ transfected cells were seeded in each well of an eight-well chambered cover glass (Thermo Fisher Scientific) the day before the Ca^2+^ imaging assay. Then, 5 μm Fluo-8 (AAT Bioquest) dissolved in HHBS solution was incubated with the cells for 30 min in a cell incubator and 30 min at room temperature after removing the culture medium. These cells were washed three times with HHBS, and 360 µl HHBS was added. Subsequently, images of these cells were captured by a Zeiss 710 nonlinear optical confocal microscope before and after being treated with 10× compounds or HHBS solution.

The Ca^2+^ imaging assay was performed using an EnVision 2104 Multilabel Reader (PerkinElmer; excitation, 485 nm and emission, 535 nm). A total of 6 × 10^4^ cells grown in each well overnight on a 96-well optical-bottom plate with a cover glass base (Corning) were measured by a Fluo-8 No Wash Calcium Assay Kit (AAT Bioquest), which is the most effective dye for detecting intracellular free Ca^2+^. Cells were cultured at 37°C, and 5% CO_2_ in DMEM/F-12 medium (Thermo Fisher Scientific) containing 10% FBS (Natocor Industria Biológica) and washed with DMEM/F-12 medium once after overnight incubation.

#### β-arrestin recruitment assay

For the β-arrestin2 recruitment assay, a PRESTO-Tango assay was performed as described previously (Kroeze et al., 2015) with some modifications. HTLA cells (a HEK293 cell line stably expressing a tTA-dependent luciferase reporter and a β-arrestin2-TEV fusion gene; a gift from the laboratory of Richard Axel and Wang Sheng) grown in each well of a six-well plate were transfected with 1 µg GPCR CDS containing the Tango construct according to instructions from the manufacturer. The transfected cells were seeded in poly-l-lysine–coated 384-well plates (catalog #J03841, JingAn Biological) at 15,000–20,000 cells per well in 50 µl medium for 24 h. The medium was replaced by a fresh medium containing a series of concentrations of compounds after 24 h of culture in a 384-well plate. Medium and drug solutions were removed from each well after ∼18 h of incubation with cells, and 20 µl per well of Bright-Glo solution (Promega) was diluted 20-fold in assay buffer (1× HBSS, 2.5 mm probenecid, 20 mm HEPES, pH 7.4) and added to each well. After ∼15 min of incubation at room temperature, luminescence was quantified in an EnVision 2104 Multilabel Reader (PerkinElmer) or FlexStation 3 system (Molecular Devices). The relative luminescence was exported into an Excel sheet and processed with Excel and GraphPad Prism 6 software. Procedures for the β-arrestin1/2 recruitment assay were the same, except that 0.5 µg β-arrestin1-expressing plasmid was transfected together with 1 µg Tango-GPCR plasmids into HTLA cells.

### Quantification, statistical analysis, and reproducibility

We performed quantification using a minimum of three independent biological replicates. Data collection and analysis were not performed in a blinded manner if not specified, but different research groups performed the quantifications. We present group data as the mean ± SEM. We used IBM SPSS 25.0 software and Microsoft Excel to perform statistical analyses, including Student's unpaired *t* tests, Student's paired *t* tests, one-way ANOVA with Tukey's *post hoc* tests, and two-way repeated-measures (RM) ANOVA with Tukey's HSD. Asterisks or number signs denote significant differences in all figures (*p < 0.05, *p < 0.01, ***p < 0.001, ****p < 0.0001, and ####*p* < 0.0001). N.S. indicates a nonsignificant difference. We used OriginPro 8.5 (OriginLab Corporation), Inkscape 0.92.3 (Inkscape), and GraphPad Prism 6 software to create graphs. The reference brain map with anatomic abbreviations is accessible on the Allen Brain Atlas website.

## Results

### CCK-INs potentiate inhibition in the AC

We adopted an *in vivo* recording animal model for the first experiment. Considering activation of GABAergic neurons induces inhibitory outputs to nearby neurons in the neocortex, we assumed prior activation of GABAergic neurons would suppress neuronal responses in the AC to a forthcoming AS.

**Figure 1.**
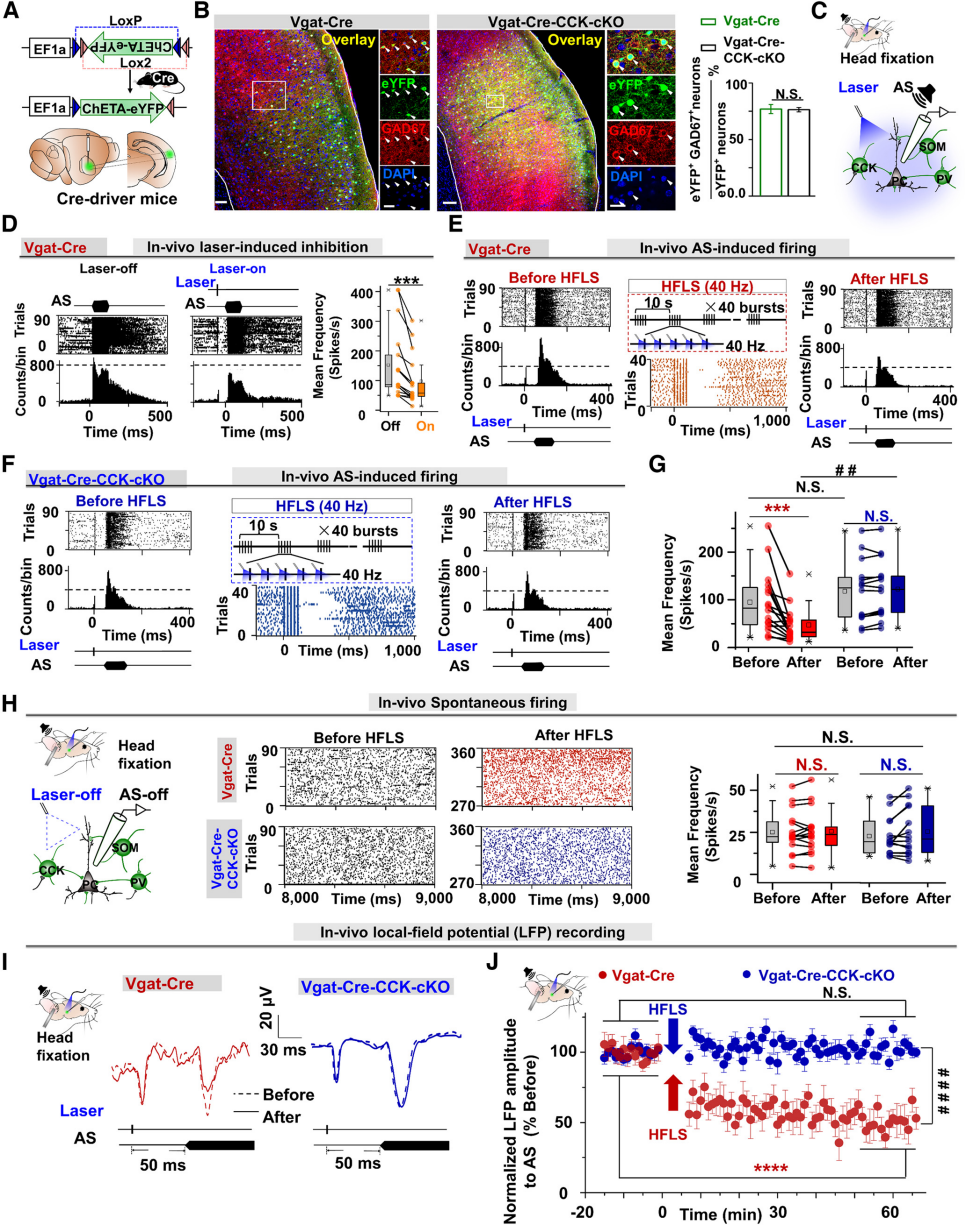
HFLS of GABAergic CCK neurons enhances their inhibition in the long term. ***A***, Schematic illustration of Cre-dependent AAV vector (DIO-ChETA-eYFP) injection into the AC of Vgat-Cre and Vgat-Cre-CCK-cKO mice to specifically infect GABAergic neurons. ***B***, Immunohistochemical labeling images of eYFP, GAD67, and DAPI in the AC of DIO-ChETA-eYFP-injected Vgat-Cre and Vgat-Cre-CCK-cKO mice. Scale bars: 100 and 20 µm, respectively. The bar chart depicts the percentage of colabeled neurons positive for eYFP and GAD67 in virus-injected Vgat-Cre (*n* = 12 sections, *N* = 5 mice) and Vgat-Cre-CCK-cKO mice (*n* = 11 sections, *N* = 5 mice; one-way ANOVA with Tukey's *post hoc* test; N.S., not significant). ***C***, The setup for *in vivo* recordings with laser stimulation in the AC (natural AS, a noise burst; SOM, PV, and PC). ***D***, Left to right, Unit response in raster displays and PSTHs to the AS with the laser off (left) or on (right), and group data for mean frequency of the AS-triggered firing rate with the laser off or on (DIO-ChETA-eYFP-injected mice, *n* = 17 units, *N* = 11 mice; two-way ANOVA with Tukey's *post hoc* test, ***p < 0.001). ***E***, Multiunit recording in Vgat-Cre mice. Left, Unit response (middle) and mean firing rate (right) of responses to the combined stimulus of the laser and AS before, during, and after HFLS in Vgat-Cre mice. ***F***, Multiunit recording in Vgat-Cre-CCK-cKO mice. Left to right, Unit response and mean firing rate (right) for responses to the combined stimulus of the laser and AS before, during, and after HFLS in Vgat-Cre-CCK-cKO mice. ***G***, Group data for ***E***, ***F*** [Vgat-Cre (red), *n* = 16 units, *N* = 11 mice; Vgat-Cre-CCK-cKO (blue), *n* = 14 units, *N* = 11 mice; two-way ANOVA with Tukey's *post hoc* test; ***p < 0.001, N.S.; Vgat-Cre vs Vgat-Cre-CCK-cKO, two-way ANOVA with Tukey's *post hoc* test, ##*p* < 0.01]. ***H***, Unit response and mean spontaneous firing rate before and after HFLS in DIO-ChETA-eYFP–injected Vgat-Cre (*n* = 16 units, *N* = 11 mice) and Vgat-Cre-CCK-cKO mice (*n* = 14 units, *N* = 11 mice; two-way ANOVA with Tukey's *post hoc* test, N.S.). ***I***, LFP response to the AS (with a preceding laser pulse). Representative traces before (dashed lines) and after (solid lines) HFLSs were recorded in Vgat-Cre (red) and Vgat-Cre-CCK-cKO (blue) mice. ***J***, Time course of the normalized amplitude of LFP responses to the AS (with a preceding laser pulse) before and after HFLS [Vgat-Cre-CCK-cKO (blue), *n* = 16 recording sites, *N* = 11 mice; Vgat-Cre (red), *n* = 14 recording sites, *N* = 11 mice, two-way RM ANOVA with Tukey's *post hoc* test, ****p < 0.0001, ####*p* < 0.0001, N.S., not significant]. Data indicate mean ± SEM.

Conditional CCK-KO (CCK-cKO) mice were generated using the CRISPR/Cas9 system. Vgat-Cre and CCK-cKO mice were crossed to purposefully knock out CCK in GABAergic neurons (CCK^−/−^-GABA). We injected the same Cre-dependent adeno-associated virus (AAV) vector AAV-EF1a-DIO-ChETA-eYFP in the AC of Vgat-Cre and Vgat-Cre-CCK-cKO mice (Fig. 1*A*) to infect GABAergic neurons specifically. Most eYFP^+^ neurons colocalized with GAD67 (Fig. 1*B*; Vgat-Cre, 76.91 ± 4.03%, *n* = 12 sections, *N* = 5 mice; Vgat-Cre-CCK-cKO, 76.17 ± 1.92%, *n* = 11 sections, *N* = 5 mice, one-way ANOVA with Tukey's *post hoc* test, N.S.).

In the *in vivo* setup for anesthetized mice (Fig. 1*C*), normalized sound intensity, and laser power were selected after measuring the input-output curve of each mouse for stimulus intensities. Also, the kinetics of optogenetic opsin were examined by applying laser pulse trains of different frequencies (1–120 Hz) in both groups to ensure normal firing of GABAergic neurons after knocking out CCK.

Laser stimulation significantly suppressed evoked response to the forthcoming AS in ChETA-eYFP-injected Vgat-Cre mice (Fig. 1*D*, left), response to the AS in raster displays (90 trials over 15 min), and poststimulus histograms (PSTHs; bin width, 10 ms; middle, response to the AS preceded by laser stimulation; right, mean auditory response calculated in a 100 ms observation window starting 20 ms after the AS in ChETA-eYFP-injected mice, which was reduced from 152.23 ± 29.22 to 104.07 ± 20.75 Hz (*n* = 17 units; two-way ANOVA with Tukey's *post hoc* test, ***p < 0.001). Activation of local GABAergic neurons in the AC inhibited responses to the forthcoming natural AS in the AC.

Next, we examined whether HFLS of GABAergic neurons could potentiate their inhibition of neuronal responses to the forthcoming AS in the AC and whether CCK-INs are involved in potentiating their inhibitory outputs toward their target neurons.

Laser-activated GABAergic neurons showed inhibited neuronal responses to the forthcoming AS in Vgat-Cre mice (Fig. 1*E***–***F*). We adopted the HFLS protocol (five-pulse bursts at 40 Hz, 10 s interburst interval, 200 pulses in total). HFLS of GABAergic neurons potentiated the inhibitory effect of laser-activated GABAergic neurons on neuronal responses to the forthcoming AS in Vgat-Cre mice (Fig. 1*E*, from left to right, before and after HFLS and group data for firing rate in raster displays and PSTHs). We found no potentiation of inhibition in Vgat-Cre-CCK-cKO mice, which showed similar laser-evoked responses as Vgat-Cre mice before, during, and after HFLS [Fig. 1*F*, from left to right, rasters before, during, and after HFLS in Vgat-Cre-CCK-cKO mice; Fig. 1*G*, group firing rates before and after HFLS, Vgat-Cre mice (red), 113.08 ± 36.92 and 63.86 ± 17.82 Hz, *n* = 16 units, *N* = 11 mice, two-way ANOVA with Tukey's *post hoc* test, ***p < 0.001; Vgat-Cre-CCK-cKO (blue), 116.63 ± 17.79 and 120.97 ± 16.98 Hz, *n* = 14 units, *N* = 11 mice; two-way ANOVA with Tukey's *post hoc* test; N.S.; Vgat-Cre, 63.86 ± 17.82 Hz vs Vgat-Cre-CCK-cKO, 120.97 ± 16.98 Hz, two-way ANOVA with Tukey's *post hoc* test, ##*p* < 0.01]. The spontaneous firing rates of the two groups remained the same before and after HFLS (Fig. 1*H*). These results suggest enhanced inhibition occurred when neuronal responses exceeded a certain excitatory threshold.

The enhanced inhibition was also reflected in LFP responses. Example traces in Figure 1*I* and group data in Figure 1*J* show that HFLS induced a distinct reduction in the amplitude of LFPs to the AS in Vgat-Cre mice but not in Vgat-Cre-CCK-cKO mice (Fig. 1*J*; Vgat-Cre, 52.75 ± 6.84% vs Vgat-Cre-CCK-cKO, 103.13 ± 2.34%, two-way RM ANOVA with Tukey's *post hoc* test, ####*p* < 0.0001).

In summary, HFLS of local GABAergic neurons potentiated the inhibitory effect of GABAergic neurons on neuronal responses to the forthcoming AS in Vgat-Cre mice but not in Vgat-Cre-CCK-cKO mice. The potentiated inhibition was long lasting and persisted across the observation period of 60 min (Fig. 1*J*).

### HFLS of CCK-INs, but not PV-INs, potentiates sIPSCs in glutamatergic neurons

Earlier studies on cortical inhibitory neuron-related plasticity and behaviors extensively focus on discriminating the distinct roles of PV-IN, SOM-IN, and VIP-IN-induced inhibitory control (Kvitsiani et al., 2013; Pfeffer et al., 2013; Pi et al., 2013; Chen et al., 2017; Yu et al., 2019; Jang et al., 2020) because they account for the majority of neocortical GABAergic neurons (Gonchar, 2008; Whissell et al., 2015) and are easily characterized by selective genetic and histologic labeling. In contrast, cortical CCK-INs constitute a major subclass of GABAergic interneurons but receive less attention because of difficulties isolating CCK-INs.

The above *in vivo* experiments show that HFLS of GABAergic neurons in Vgat-Cre mice but not in Vgat-Cre-CCK-cKO mice potentiated the inhibition of neuronal activity in the AC. In the next experiment, we performed a patch-clamp recording of pyramidal neurons to examine whether the potentiated inhibition of GABAergic inputs was CCK-IN specific. We targeted CCK-INs and considered PV-INs as a control as PV-INs represent the largest proportion of GABAergic neurons in the cortex and hippocampus and serve to gate perisomatic inhibition (Gonchar, 2008; Whissell et al., 2015; Dudok et al., 2021). Dudok et al. (2021) specifically labeled hippocampal CA1 CCK basket cells Sncg (58 ± 17%), but no other inhibitory subtypes using the Sncg-Flp mouse line (Dudok et al., 2021). To unbiasedly label cortical CCK-INs and PV-INs, we combined a Cre recombinase-based genetic approach with the inhibitory neuron-specific intergenic regulatory sequence Dlx5/6 (Whissell et al., 2015), which provides selective genetic access and sufficient labeling in available PV-Cre and CCK-Cre transgenic mouse lines (Taniguchi et al., 2011).

**Figure 2.**
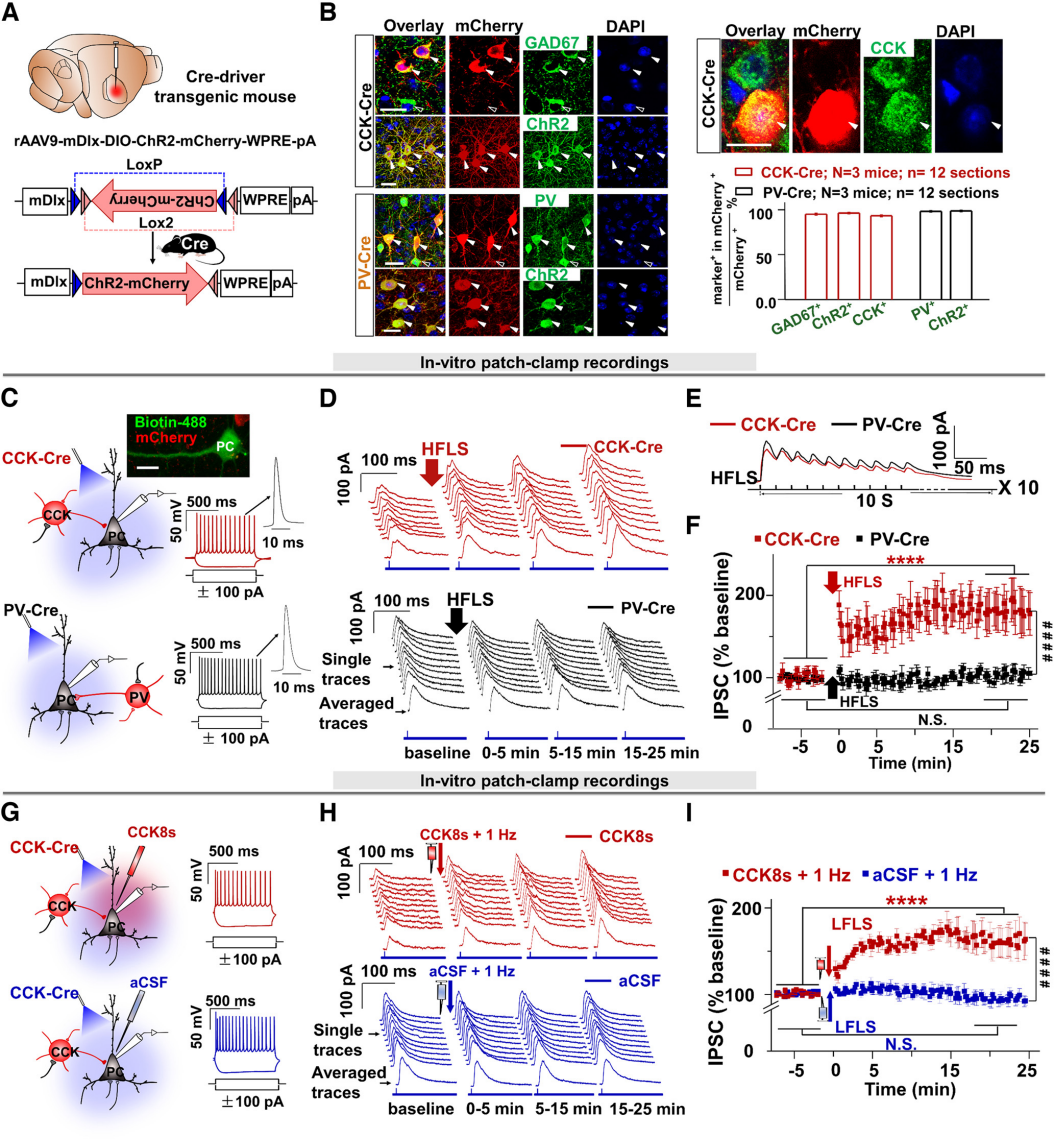
HFLS of GABAergic CCK synapses potentiates IPSCs in postsynaptic glutamatergic neurons. ***A***, Injection of rAAV9-mDlx-DIO-ChR2-mCherry into CCK-Cre and PV-Cre mice. ***B***, Images for mCherry colabeled with the indicated markers (GAD67, ChR2, PV, and CCK) in the AC of CCK-Cre or PV-Cre mice. Scale bars: 20 µm. The bar chart depicts the percentage of mCherry^+^ neurons colabeled with GAD67, ChR2, and CCK in CCK-Cre mice or colabeled with PV and ChR2 in PV-Cre mice. ***C***, Schematic drawings show *in vitro* patch-clamp recording of target PC neurons while delivering a laser pulse to activate infected CCK neurons (top) and PV neurons (bottom). The confocal image depicts the morphology of a representative pyramidal neuron infused with Alexa Flour 488 after patch-clamp recording in CCK-Cre mice. Scale bar, 15 µm. Neuronal firing responses to step currents show the recruited PC with regular spiking. ***D***, Traces of IPSC responses to the laser pulse at 0.1 Hz in brain slices from CCK-Cre and PV-Cre mice before and after HFLS. ***E***, Sampled neuronal responses to the HFLS in PV-Cre and CCK-Cre brain slices. ***F***, Time course of normalized IPSCs before (for 5 min) and after (for 25 min) HFLS in PV-Cre (black, *n* = 9 neurons; one-way ANOVA with Tukey's *post hoc* test; N.S.) and CCK-Cre (red, *n* = 9 neurons; one-way ANOVA with Tukey's *post hoc* test, ****p < 0.0001; two-way RM ANOVA with Tukey's *post hoc* test, ####*p* < 0.0001) mice. ***G***, Schematic drawings show patch-clamp recording targeted at pyramidal neurons while delivering a laser pulse to activate infected neurons and perfusing CCK8s or aCSF. Neuronal responses to step currents were used to confirm that recorded neurons were excitatory with regular spike trains as in **C**. ***H***, IPSCs in response to the laser pulse in brain slices from CCK-Cre mice perfused with CCK8s or aCSF. ***I***, Time course of normalized IPSCs before (for 5 min) and after (for 25 min) LFLS in brain slices from CCK-Cre mice perfused with aCSF (blue, *n* = 8 neurons; one-way ANOVA with Tukey's *post hoc* test, N.S., not significant) or CCK8s (red, *n* = 8 neurons; one-way ANOVA with Tukey's *post hoc* test, ****p < 0.0001; two-way RM ANOVA, *post hoc* with Tukey HSD, ####*p* < 0.0001). Data are expressed as mean ± SEM.

To virally target PV-INs or CCK-INs to a comparable extent, we injected the Cre-dependent AAV virus into the AC of PV-Cre or CCK-Cre mice. We designed a Channelrhodopsin-2 (ChR2) and mCherry-expressing AAV under the control of the inhibitory neuron-specific Distal-less homeobox (mDlx) gene enhancer (rAAV9-mDlx-DIO-ChR2-mCherry-WPRE-pA, hereon referred to as mDlx-DIO-ChR2-mCherry; Fig. 2*A*). mDlx enhancer expression is specific to and robust in cortical interneurons across vertebrate species (Dimidschstein et al., 2016). Our virus labeled mostly CCK-INs or PV-INs in CCK-Cre or PV-Cre mice, respectively (Fig. 2*B*; 96.3% of mCherry-labeled neurons were GAD67^+^, 95.4 ± 1.6% were CCK^+^, and 96.7 ± 1.2% were ChR2^+^ in CCK-Cre mice; 97.5 ± 1.0% of mCherry-labeled neurons were PV^+^ and 98.2 ± 1.0% were ChR2^+^ in PV-Cre mice).

Brain slices from CCK-Cre and PV-Cre mice were used for *in vitro* patch-clamp recording (Fig. 2*C*). First, we selectively patched pyramidal neurons. We chose pyramidal-shaped cells that emitted regular spike trains in response to current-step injection, as in previous studies (Connors and Gutnick, 1990; Stiefel et al., 2013). In Figure 2*C*, the morphology of the pyramidal cell was visualized by Biotin 488. The physiological properties were reflected by the responses to injected step currents, after which IPSCs (with a short latency of 4.53 ± 0.28 ms, *n* = 10 cells) evoked by laser stimulation (0.15 Hz, 5 min as baseline) were directly recorded. IPSCs in CCK-Cre mice were potentiated robustly after HFLS, whereas no potentiation of IPSCs occurred in PV-Cre mice (Fig. 2*D*; single, and averaged traces of IPSCs in response to laser stimulation at 0.15 Hz before and after HFLS during 0–5 min, 5–15 min, and 15–25 min recording periods; Fig. 2*E*, representative neuronal responses to HFLS). The time course of normalized IPSCs before (for 5 min) and after (for 25 min) HFLS showed no significant potentiation of IPSCs in PV-Cre mice (Fig. 2*F*; baseline vs 20–25 min after HFLS, 100.00 ± 2.43% vs 103.67 ± 7.00%, *n* = 7 neurons, two-way ANOVA with Tukey's *post hoc* test, *p* = 0.26, N.S.), whereas HFLS of CCK-INs induced significant potentiation of IPSCs in CCK-Cre mice (Fig. 2*F*; baseline vs 20–25 min after HFLS, 101.56 ± 5.73% vs 183.70 ± 6.25%, *n* = 9 neurons, one-way ANOVA with Tukey's *post hoc* test, ****p < 0.0001).

In summary, HFLS of CCK-INs induced significant potentiation of IPSCs during the recording period, or iLTP (HFLS of CCK-INs vs HFLS of PV-INs, 183.70 ± 6.25% vs 103.67 ± 7.00%, two-way RM ANOVA with Tukey's *post hoc* test, ####*p* < 0.0001).

### Application of CCK8s and lLFLS to CCK-INs induces iLTP in glutamatergic neurons

Next, we hypothesized that HFLS of CCK-INs triggers the corelease of GABA and CCK that induces the iLTP of their target neurons. At the same time, LFLS with the same number of pulses causes no CCK release and, thus, no iLTP. In this case, the direct application of CCK followed by LFLS could generate iLTP in CCK-INs.

To test this hypothesis, we adopted the same *in vitro* patch-clamp recording method in pyramidal neurons in the AC with CCK-INs selectively transfected with AAV virus (mDlx-DIO-ChR2-mCherry; Fig. 2*G*). We applied CCK8s or aCSF followed by LFLS (Fig. 2*H*). We observed large iLTP in the CCK8s + 1 Hz group and no iLTP in the aCSF + 1 Hz group (Fig. 2*H*, single and averaged traces of IPSCs; Fig. 2*I*, normalized IPSC amplitudes; baseline vs 20–25 min after CCK8s + 1 Hz, 100.00 ± 2.53% vs 161.84 ± 4.38%, *n* = 8 neurons; baseline vs 20–25 min after aCSF + 1 Hz, 100.10 ± 1.96% vs 92.05 ± 6.58%, *n* = 7 neurons, two-way ANOVA with Tukey's *post hoc* test, ****p < 0.0001; N.S.).

Therefore, iLTP in CCK-INs was induced by the application of CCK8s followed by LFLS but not in the aCSF control group (20–25 min after aCSF + 1 Hz vs 20–25 min after CCK8s + LFLS, two-way RM ANOVA with Tukey's *post hoc* test, ####*p* < 0.0001).

### HFLS induces iLTP in CCK1R/2R-KO mice

Next, we aimed to determine which CCK receptor mediates iLTP in CCK-INs. There are two known CCK receptors in the brain, CCK1R and CCK2R. CCK2R is widely distributed throughout the CNS, whereas CCK1R is only found in certain regions, such as the hypothalamus and hippocampus (Honda et al., 1993). We reported that CCK2R mediates the role of entorhinal-cortical CCKs in cortical LTP and the encoding of associative memory (Li et al., 2014; Chen et al., 2019). Thus, we expected that CCK-induced iLTP is mediated by CCK receptors.

We used CCK1R/2R-KO and CCK-KO mice to examine which CCK receptor mediates the contribution of CCK signaling to the iLTP of CCK-INs in brain slice experiments. We performed genotyping confirmation of CCK1R/2R-KO and CCK-KO mouse lines before virus injection and *in vitro* recording.

**Figure 3.**
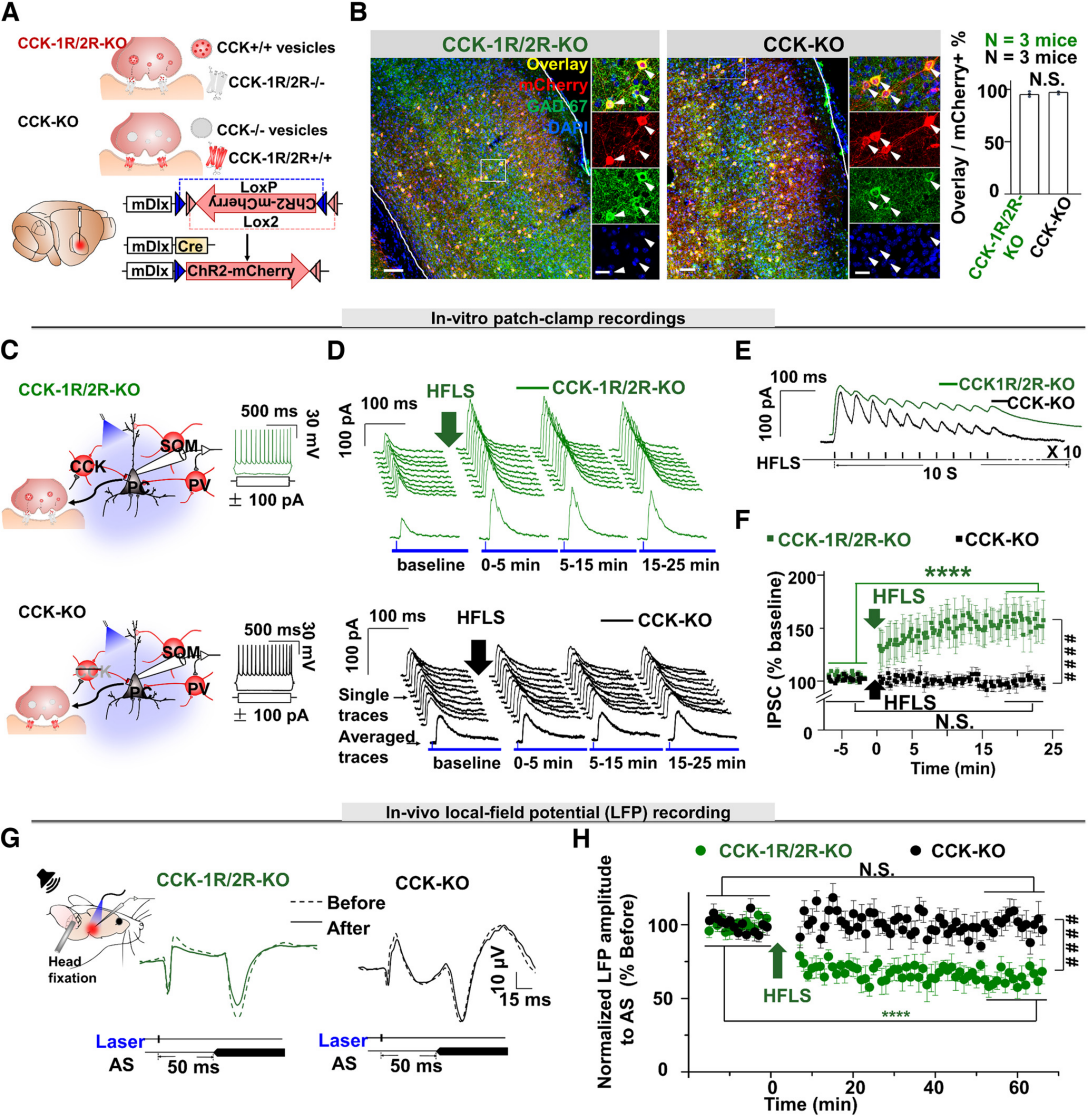
Known CCK receptors do not mediate CCK signaling in CCK-INs. ***A***, Schematic drawings show rAAV9-mDlx-DIO-ChR2-mCherry-WPRE-pA (mDlx-DIO-ChR2-mCherry) and AAV9-mDlx-Cre-WPRE-pA (mDlx-Cre) injected into CCK1R/2R-KO or CCK-KO mice. ***B***, AAV9-infected mCherry neurons colabeled with GAD67 (left, CCK1R/2R-KO mouse; right, CCK-KO mouse). Scale bars: 50 and 10 µm, respectively. Right, Bar chart shows the percentage of mCherry neurons colabeled with GAD67 in CCK1R/2R-KO and CCK-KO mice. ***C***, Schematic drawings show *in vitro* patch-clamp recordings from pyramidal neurons while delivering a laser pulse to activate infected GABAergic neurons in CCK-KO and CCK1R/2R-KO mice. Neuronal responses to a step current confirmed that the recorded neurons were excitatory. ***D***, Traces of IPSCs in response to the laser pulse in CCK1R/2R-KO (top) and CCK-KO (bottom) mice before and after HFLS of infected GABAergic neurons. ***E***, Sampled neuronal responses to HFLS in CCK1R/2R-KO and CCK-KO mice. ***F***, Time course of normalized IPSCs before and after (for 25 min) HFLS in CCK-KO (black, *n* = 9 neurons; one-way ANOVA with Tukey's *post hoc* test, N.S., not significant) and CCK1R/2R-KO mice (green, *n* = 9 neurons; one-way ANOVA with Tukey's *post hoc* test, ****p < 0.0001; two-way RM ANOVA with Tukey's *post hoc* test, ####*p* < 0.0001). ***G***, Schematic illustration of the setup for *in vivo* recordings with laser stimulation in the AC and a natural AS (a noise burst; SOM, PV neuron, PC) in CCK1R/2R-KO (left) and CCK-KO (right) mice. Bottom, Example waveforms from the two groups. ***H***, Normalized LFP response amplitudes to the AS with a preceding laser pulse before and after HFLS in CCK1R/2R-KO (green, *n* = 7 recording sites, *N* = 5 mice) and CCK-KO (black, *n* = 8 recording sites, *N* = 6 mice; two-way RM ANOVA with Tukey's *post hoc* test, ****p < 0.0001) mice. Data are expressed as mean ± SEM.

We combined two viruses (Fig. 3*A*; mDlx-DIO-ChR2-mCherry and mDlx-Cre-helper rAAV9) that both contain mDlx and are expressed only in interneurons. The viruses were injected into the AC of CCK1R/2R-KO and CCK-KO mice.

Immunohistochemical experiments examined whether the combined viruses were expressed in the target GABAergic neurons effectively and precisely. Confocal images showed that almost all infected neurons were GABAergic neurons in both mouse lines (Fig. 3*B*; one-way ANOVA with Tukey's *post hoc* test, N.S.).

In the following experiments, pyramidal neurons were selected for recording using *in vitro* patch clamping (Fig. 3*C*; preparation for recording and responses to current injection). HFLS of GABAergic neurons induced iLTP in recorded pyramidal neurons in CCK1R/2R-KO mice but not in CCK-KO mice (Fig. 3*D*, single and averaged traces; Fig. 3*E*, IPSCs in response to HFLS, 40 Hz, 10 pulses for both CCK1R/2R-KO and CCK-KO mice; Fig. 3*F*, CCK1R/2R-KO mice, baseline vs 20–25 min after HFLS, 100.36 ± 2.91% vs 150.34 ± 5.32%, *n* = 9 neurons, two-way ANOVA with Tukey's *post hoc* test, ****p < 0.0001; CCK-KO mice, baseline vs 20–25 min after HFLS, 99.93 ± 2.22% vs 97.71 ± 2.98%, *n* = 9 neurons, two-way ANOVA with Tukey's *post hoc* test, *p* = 0.82, N.S.).

We then decided to confirm these iLTPs using the same protocol for *in vivo* recording in CCK1R/2R-KO mice (Fig. 3*G*). We examined whether laser activation of GABAergic neurons suppresses neuronal responses in the AC to a forthcoming AS. As expected, HFLS of GABAergic neurons did not reduce the amplitude of LFPs in response to the AS in CCK-KO mice. To our surprise, HFLS of GABAergic neurons induced a distinct decrease in the amplitude of LFPs in response to the AS in CCK1R/2R-KO mice (Fig. 3*H*; CCK1R/2R-KO, 63.56 ± 5.09% vs CCK-KO, 100.17 ± 4.20%, two-way RM ANOVA with Tukey's *post hoc* test, ####*p* < 0.0001), indicating that a novel CCK receptor other than CCK1R or CCK2R is likely involved in mediating the signaling of CCK from CCK-INs.

These results indicate that CCK-enabled iLTP is likely mediated by a novel receptor other than CCK1R or CCK2R.

### Bioinformatics and binding assay search for potential CCK receptors

Currently, five categories of methods are mainly being used to characterize the binding of GPCRs and ligands based on their detection mechanisms, namely, direct binding assay (Stoddart et al., 2015; Flanagan, 2016; Rice et al., 2019), G-protein-based signal detection (Seifert et al., 1999; Inoue et al., 2012; Ma et al., 2017), arrestin-dependent signal detection (Bassoni et al., 2012), biophysical methods (Navratilova et al., 2011; Schroder et al., 2011; Rajarathnam and Rosgen, 2014), and bioinformatics prediction (Kharkar et al., 2014; Foster et al., 2019).

The downstream signals of many GPCRs are unclear, especially for >100 orphan GPCRs (Laschet et al., 2018). Bioinformatics prediction is the most efficient method of finding GPCR-ligand pairs and hence was adopted in our study to search for novel CCK receptors in the brain. Combining cell-based assays with bioinformatics prediction has enabled the identification of many GPCR-ligand pairs, such as bombesin receptor 3 (BB3)-neuromedin B, BB3-gastrin-releasing peptide, GPR15-GPR15L_47-57_, GPR68-Osteocrin_33-55_, and GABA_B_R1a-sAPPa (Foster et al., 2019; Rice et al., 2019). In addition, loss-of-function and gain-of-function studies coupled with histology verification are very important for exploring the physiological functions of novel GPCRs.

**Figure 4.**
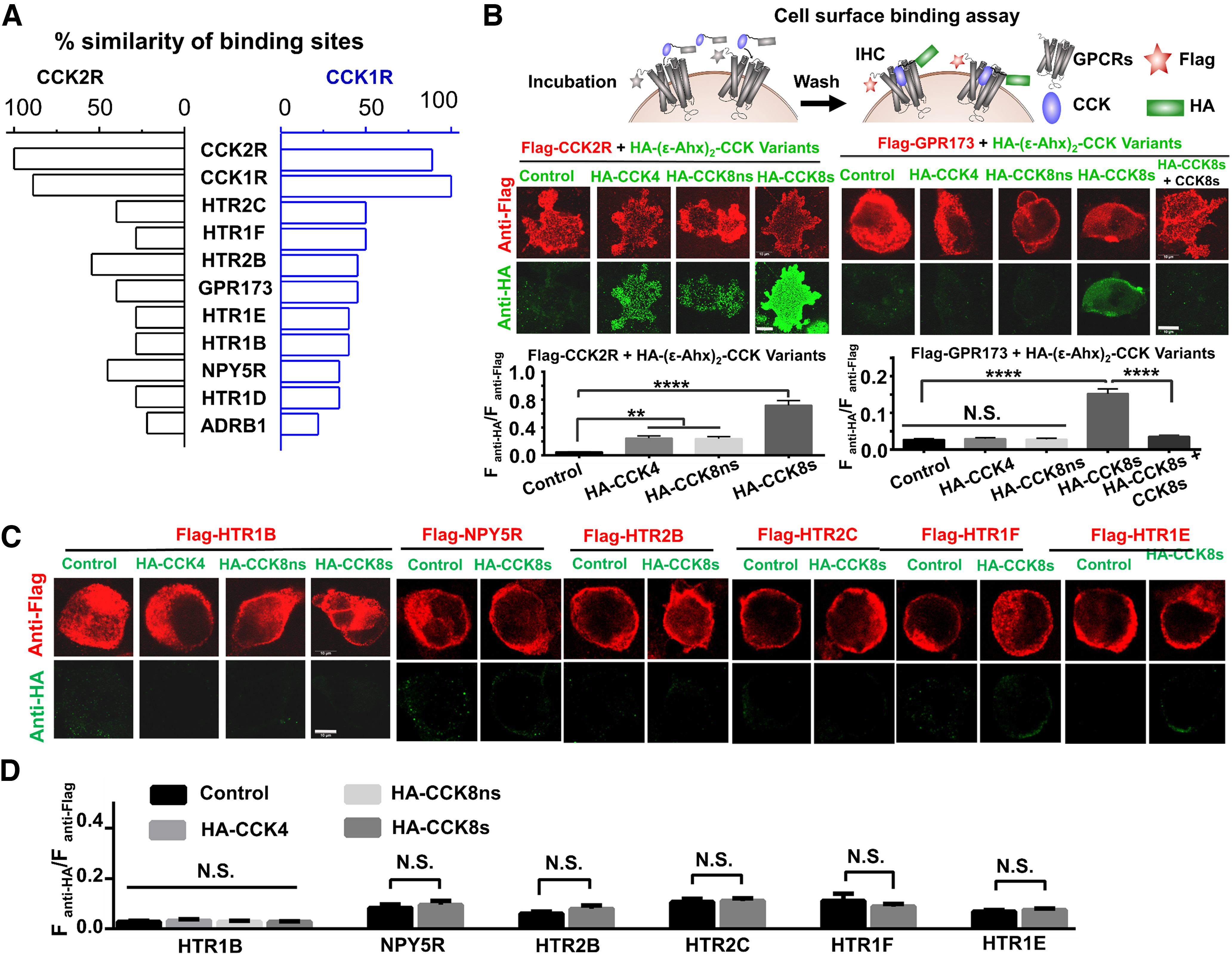
Bioinformatics and cell surface binding assay screening for a novel CCK receptor. ***A***, Bar chart showing the highest similarity scores between GPCRs and known CCK receptors (CCK1R and CCK2R) considering the secondary structure in amino acid sequences, including six GPCRs for 5-hydroxytryptamine (serotonin; HTR2C, HTR1F, HTR2B, HTR1E, HTR1B, and HTR1D), an orphan receptor (GPR173), neuropeptide Y receptor Y5 (NPY5R), and adrenergic receptor beta 1 (ADRB1). ***B–D***, Schematic of cell surface binding assay for Flag-tagged GPCRs with negative control (HA-(ε-Ahx)_2_) or HA-(ε-Ahx)_2_-CCK variants (HA-CCK4, HA-CCK8ns, and HA-CCK8s). Confocal images (***B***, top; scale bar, 10 µm) and quantification of fluorescence intensity ratio (F_anti-HA_/F_anti-Flag_; ***B***, ***D***, bottom) in the cell surface binding assay for Flag-CCK2R, Flag-GPR173, Flag-HTR1B, and five other Flag-GPCRs as shown in ***C***. The number of total cells from three independent experiments is defined as *n*; *n* = 33–40, one-way ANOVA with Tukey's *post hoc* test or unpaired Student's *t* test, **p < 0.01, ****p < 0.0001, N.S., not significant. Data are expressed as mean ± SEM.

Recently, highly accurate protein 2D alignment or 3D structure prediction has facilitated the development of new drugs targeting GPCRs (Insel et al., 2019; Yang et al., 2021). The online alignment of the 2D protein sequence (Sievers et al., 2011) and prediction of the 3D structure by AlphaFold2 or ColabFold systems (Senior et al., 2020; Du et al., 2021; Tunyasuvunakool et al., 2021; Mirdita et al., 2022; Varadi et al., 2022) enables us to map CCK receptors rapidly and comprehensively. We speculated that a new CCK receptor mediates CCK8s-induced iLTP in the mouse AC, which is probably a GPCR similar to CCK1R/2R. The 2D sequences of CCK receptors should be evolutionarily homologous. We predicted the secondary structure of amino acid sequences of selected brain GPCRs using the free online software NetSurfP version 1.1 (Petersen et al., 2009). The binding sites of all GPCR secondary structures (extramembrane) were selected based on the CCK binding domains of CCK1R and CCK2R, and the GPCRs were compared with the two CCK receptors based on hamming distance. We listed the GPCRs with the highest scores (Fig. 4*A*) as probable candidates for CCK receptors.

To further evaluate which of these GPCR candidates is a novel CCK receptor, we established a cell surface binding assay according to previous reports (De Wit et al., 2013; Rice et al., 2019; Fig. 4*B*). In this system, the CDS of candidate GPCRs, as well as CCK2R with an N-terminal Flag tag, were cloned into pCDNA3.1(+) to transfect HEK293T cells (Fig. 4*B–D*). We also synthesized a series of HA-(ε-Ahx)_2_-CCK variant probes, including CCK4, CCK8s, nonsulfated CCK8 (CCK8ns), and control HA-(ε-Ahx)_2_, which have been detected in the body with different specificity for CCK1R/2R.

As CCK8s is the main form of CCK in the brain (Dockray et al., 1978; Agersnap et al., 2016), we applied a cell surface binding assay with HA-tagged CCK8s for the first-round screening of GPCR candidates with high scores, including Flag-tagged GPR173, HTR2C, HTR1F, HTR2B, HTR1E, HTR1B, and NPY5R (Fig. 4*B*,*C*). We demonstrated that HA-tagged CCK4, HA-tagged CCK8ns, and HA-tagged CCK8s colocalized with Flag-tagged CCK2R, with a significantly larger ratio of CCK ligand HA-tag signal to CCK2R Flag signal (F_anti-HA_/F_anti-Flag_) than that of the HA-(ε-Ahx)_2_ control group, suggesting that our cell surface binding assay was applicable for characterizing the binding of CCK ligands and receptors.

CCK-2R and GPR173 bound to CCK8s, whereas other GPCR candidates did not (Fig. 4*B–D*). Thus, detailed ligand specificity analysis was performed using HA-tagged CCK4/CCK8ns/CCK8s probes, in which HTR1B-expressing cells served as negative/background control. Flag-tagged GPR173 could only bind to HA-tagged CCK8 but not HA-tagged CCK4 or CCK8ns (Fig. 4*B*), indicating that CCK8s is a specific ligand of GPR173. No binding of HA-tagged CCK4/CCK8ns/CCK8s with Flag-tagged HTR1B was detected. To further verify the binding between GPR173 and CCK8s, we used a competitive cell surface binding procedure, in which Flag-tagged GPR173-expressing HEK293T cells were incubated with a CCK8s and HA-tagged CCK8s (1:1) mixture. We found that CCK8s significantly blocked the HA-tag signals from binding HA-tagged CCK8s (Fig. 4*B***–***D*). Together, we conclude that GPR173 is a novel CCK receptor, and CCK8s is a ligand of GPR173.

### GPR173 is located at CCK-GABAergic synapses in the AC

The above evidence confirmed that GPR173 is a novel CCK receptor. Here, we performed histochemical analysis and used another bioinformatics prediction algorithm to verify whether GPR173 or another novel CCK receptor mediates HFLS-induced iLTP.

**Figure 5.**
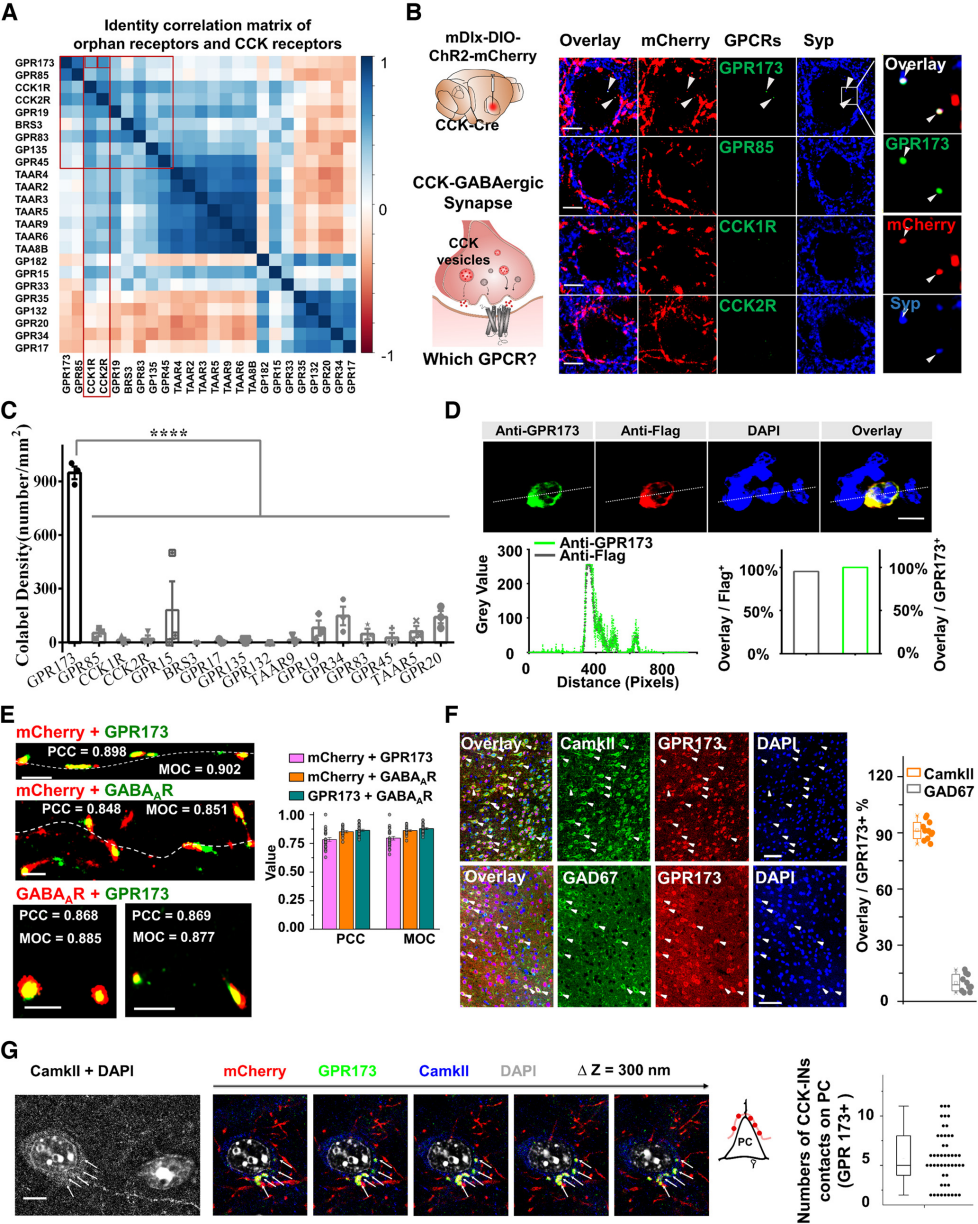
Bioinformatics and histology screening for a novel CCK receptor. ***A***, Percentage identity correlation matrix of orphan receptors with CCK1R/2R. ***B***, Left, Illustration of mDlx-DIO-ChR2-mCherry injected into the AC of CCK-Cre mice. Middle, Immunochemistry staining images of candidate receptors in CCK-GABAergic synapses. Scale bars: 5 µm. Expanded images are from the white rectangles (top right). The white arrows indicate the colocalization of GPR173 with CCK-GABAergic synapses. Scale bars: 1 µm. ***C***, Bar chart shows numbers of overlay dots [bottom right, mCherry^+^, Syp (synaptophysin)^+^, and GPCR^+^, per mm^2^; *n* = 3 independent experiments; one-way ANOVA with Tukey's *post hoc* test, ****p < 0.0001]. ***D***, Confocal images of Flag-GPR173-transfected HEK293T cells colabeled with anti-GPR173, anti-Flag, and DAPI. Scale bar, 10 µm. Quantitative analysis of colocalization of Flag and GPR173 across the dotted line (labeled in confocal images). The bar chart depicts the percentage of colabeled cells positive for Flag and GPR173 from Flag-GPR173–transfected HEK293T cells stained with anti-GPR173, anti-Flag, and DAPI (*n* = 21 cells). ***E***, Super-resolution image of colocalized GPR173 with CCK-GABAergic synapses, GABA_A_R with CCK-GABAergic synapses, and GABA_A_R and GPR173 (left). Scale bar, 1 µm. The values for PCC and MOC indicate the colocalization possibility of mCherry with GPR173, mCherry with GABA_A_R, and GABA_A_R with GPR173. ***F***, Immunochemistry images of costaining of GPR173 with CaMKII (top) or GAD67 (bottom) in C57BL/6J mice. Scale bar, 50 µm. Group data from colocalization analysis of GPR173 with CaMKII (*n* = 11 sections, *N* = 3 mice) or GAD 67 (*n* = 10 sections, *N* = 3 mice). ***G***, Immunochemistry images for colocalized GPR173 with CCK-GABAergic synapses contacting pyramidal cells and box plot showing numbers of colocalized GPR173 and CCK-GABAergic contacts per pyramidal cell (*n* = 49 neurons, 5.69 ± 0.38). Scale bar, 5 µm. Data are expressed as mean ± SEM.

In the bioinformatics prediction algorithm, we used the Clustal Omega program (Sievers et al., 2011) to extract homologous information for 79 orphan receptors from class A GPCRs aligned with the two known CCK receptors. The percentage identity correlation score defines the quantitative correlation estimation between two sets of sequences (Raghava and Barton, 2006). According to the percentage identity correlation score among 79 orphan receptors and CCK1R/2R, we selected top-scored orphan receptors normalized by CCK1R and CCK2R (Fig. 5*A*). Interestingly, GPR83, which was recently reported as a novel CCK receptor (Mack et al., 2022), was also among the five top-scored candidates (Fig. 5*A*).

The next important question was whether GPR173, GPR83, or other new CCK receptors mediate CCK-dependent iLTP. If so, the new CCK receptors should localize postsynaptically at CCK-GABAergic synapses. We injected mDlx-DIO-ChR2-mCherry AAV in the CCK-Cre mouse AC to label CCK-INs (Fig. 5*B*, left). We performed triple-labeling of mCherry, synaptophysin, and the GPCR candidates with the highest correlation scores. GPR173 had the highest colocalization density (Fig. 5*C*; GPR173 vs GPR85, the highest among other GPCRs, 948.90 ± 35.36 vs 53.46 ± 17.68 counts/mm^2^, one-way ANOVA with Tukey's *post hoc* test, ***p < 0.0001) with CCK-GABAergic synapses when compared with 15 other GPCRs—GPR83, CCK1R, CCK2R, GPR85, GPR15, BRS3, GPR17, GPR135, GPR132, TAAR9, GPR19, GPR34, GPR45, TAAR5, and GPR20—suggesting that GPR173 is the most likely CCK receptor mediating HFLS-induced iLTP. To verify the specificity of the GPR173 antibody, pCDNA3.1(+)-Flag-GPR173 was used to transfect HEK293T cells and perform immunofluorescence staining with anti-GPR173 (conjugated with goat anti-rabbit Alexa Fluor 647) and anti-Flag (conjugated with goat anti-mouse Alexa Fluor 568) antibodies (Fig. 5*D*). Most of the GPR173^+^ cells were also Flag^+^ cells (overlay cells, Flag^+^ cells = 95.23%). We applied a normal-intensity excitation laser and exposure time to both channels to avoid spectral bleed-through. Some Flag^+^ cells were not GPR173^+^ in parallel control experiments.

We also checked whether GPR173 colocalizes with GABA_A_R in mDlx-DIO-ChR2-mCherry-injected CCK-Cre mice using super-resolution imaging with a STORM confocal microscope (Fig. 5*E*). We found that both GPR173 and GABA_A_R appeared within mCherry-labeled CCK-GABAergic synapses, and GPR173 and GABA_A_R also nearly colocalized (Fig. 5*E*). To determine the co-occurrence and correlation coefficients of GPR173, GABA_A_R, and mCherry-labeled CCK-GABAergic synapses, we further analyzed data from super-resolution images using PCC and MOC, which are common metrics for measuring the predictability of colocalization (Aaron et al., 2018; Fig. 5*E*). The numerical values of PCC and MOC were >0.78 across six clusters, suggesting that GPR173 is located in GABAergic synapses and near GABA_A_Rs.

Moreover, as previous *in vivo* and *in vitro* recording studies described, HFLS-induced iLTP occurs in excitatory pyramidal neurons. We performed costaining of GPR173 with CaMKII (a marker of pyramidal neurons) or GAD67 (a marker of inhibitory neurons; Fig. 5*F*). More than 90% of GPR173^+^ cells were CaMKII^+^ neurons (91.27 ± 1.48% of GPR173^+^ cells were CaMKII^+^ neurons, *n* = 11 sections from 3 C57BL/6J wild-type mice). However, only a small number of GPR173^+^ cells were costained with GAD67 in the AC (Fig. 5*F*; 9.82 ± 1.42% were GAD67^+^, *n* = 10 sections from 3 C57BL/6J wild-type mice).

To further confirm that CCK-GABAergic synapses directly contact excitatory pyramidal neurons, we counted numbers of colocalized GPR173 and CCK-INs contacts on CaMKII^+^ neurons (Fig. 5*G*; *n* = 49 neurons, 5.69 ± 0.38) in cortical slices from CCK-Cre mice (as described in Fig. 5*B*). All these data suggest that GPR173 is located at CCK-GABAergic synapses and might be involved in CCK-dependent HFLS-induced iLTP. In the present study, we cannot exclude the possibility that CCK-IN–induced iLTP is restricted to the CCK postsynaptic area without affecting neighboring PV synapses. It would be worth investigating whether this iLTP is homosynaptic or heterosynaptic.

### CCK8s triggers intracellular Ca^2+^ mobilization through GPR173

CCK1R and CCK2R could couple to Gq protein, through which CCK1R/2Rs trigger intracellular Ca^2+^ release from the endoplasmic reticulum after their activation (Dunlop et al., 1996; Dufresne et al., 2006). CCK8s, as a potent but nonselective agonist of both CCK1R and CCK2R (Berna et al., 2007), mainly works via the Gq-PLC-IP_3_-Ca^2+^ pathway (Dunlop et al., 1996; Wu et al., 1997). We hypothesized that the novel CCK receptor should also trigger intracellular Ca^2+^ mobilization through this pathway on binding to CCK8s. Thus, we built a cell-based Ca^2+^ imaging assay by constructing stable monoclonal GPCR transgenic CHO cell lines for CHO-GPR173, CHO-CCK1R, and CHO-CCK2R.

**Figure 6.**
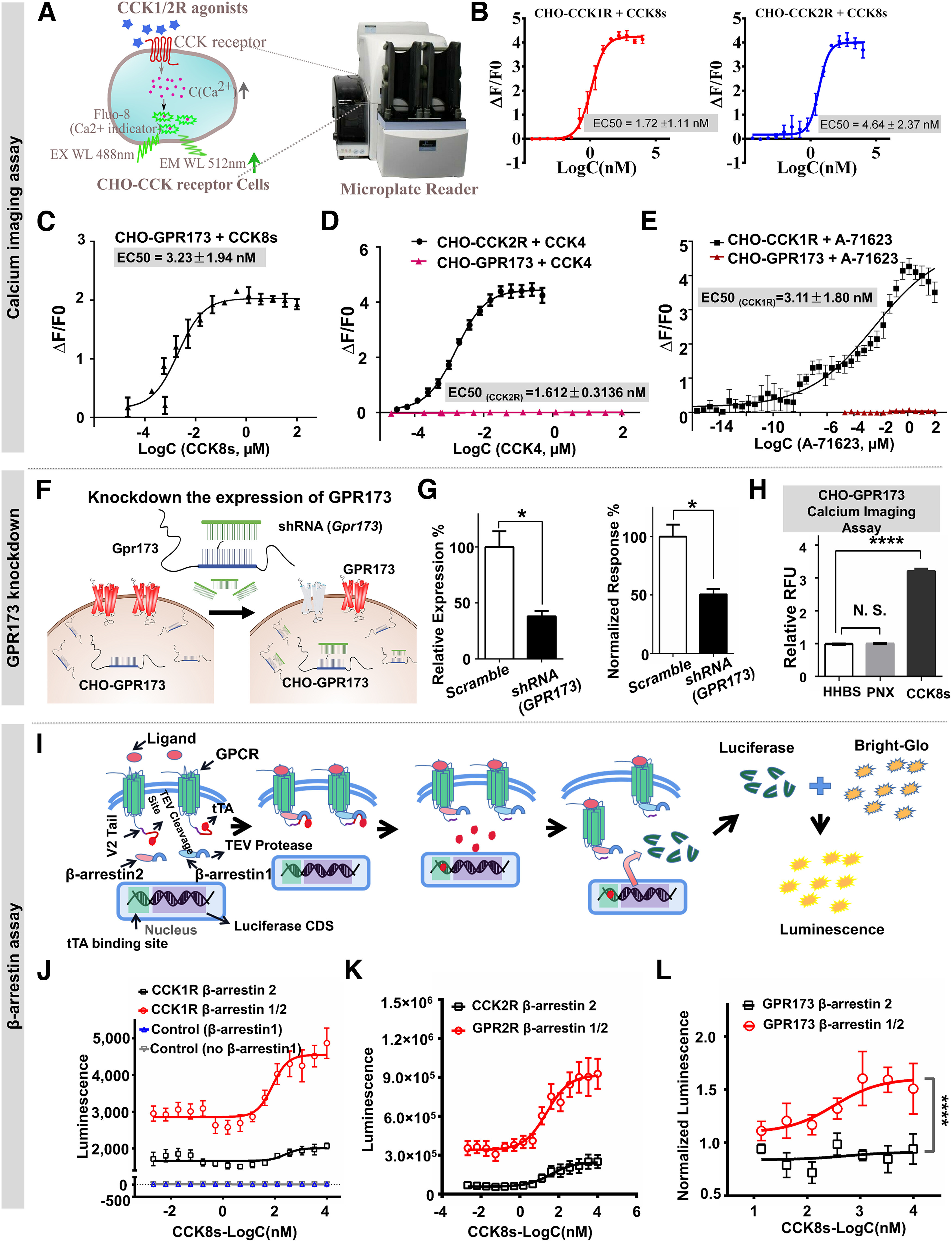
CCK8s could trigger intracellular Ca^2+^ mobilization through GPR173 in CHO-GPR173 cells. ***A***, Schematic illustration of Ca^2+^ imaging assay in CHO-GPCR cell lines. ***B***, Dose-dependent Ca^2+^ responses of CHO-CCK1R (left; EC_50_ = 1.72 ± 1.11 nm) and CHO-CCK2R (right; EC_50_ = 4.64 ± 2.37 nm) cells provoked by CCK8s. ***C***, EC_50_ curve of CCK8s for the CHO-GPR173 cell line (EC_50_ = 3.23 ± 1.94 nm). ***D***, Dose-dependent Ca^2+^ response of CHO-GPCR cells provoked by the CCK2R agonist CCK4. ***E***, Dose-dependent Ca^2+^ response of CHO-GPCR cells provoked by the CCK1R-specific agonist A-71 623. ***F***, Schematic illustration of knockdown of GPR173 expression in CHO-GPR173 cells using an shRNA of Gpr173. ***G***, 200 nm CCK8s provoked the Ca^2+^ response in the scramble or shRNA-infected CHO-GPR173 cells and Gpr173 mRNA expression level in the scramble or shRNA-infected CHO-GPR173 cells. ***H***, Ca^2+^ signal responses of CHO-GPR173 cells treated with 10 μm PNX or 200 nm CCK8s. Relative fluorescence unit (RFU) is defined as the ratio of RFU before and 30 s after adding CCK8s/HHBS (unpaired Student's *t* test, *p < 0.05,****p < 0.0001, N.S., not significant). ***I***, Illustration of the β-arrestin1/2 recruitment assay design. ***J***, Dose-dependent response of CCK8s-induced β-arrestin recruitment to CCK1Rs determined by the β-arrestin2 recruitment and β-arrestin1/2 recruitment assays. The two control groups were without GPCR plasmid transfection in HTLA cells. ***K***, ***L***, Dose-dependent response of CCK8s-induced β-arrestin recruitment to CCK2Rs (***K***) and GPR173 (***L***) determined by the β-arrestin2 recruitment and β-arrestin1/2 recruitment assays (at least *n* = 3 independent experiments; one-way ANOVA with Tukey's *post hoc* test or unpaired Student's *t* test, ****p < 0.0001). Data are expressed as mean ± SEM.

In this assay, we used a microplate reader to monitor Ca^2+^ mobilization (Fig. 6*A*). As expected, CCK8s could induce a dose-dependent response in CHO-CCK1R/2R cells (Fig. 6*B*; EC_50 CCK1R_ = 1.72 ± 1.11 nm, EC_50 CCK2R_ = 4.64 ± 2.37 nm). Then, we used the same methods for GPR173 cells and found that CCK8s triggered intracellular Ca^2+^ mobilization with a high potency of EC_50 GPR173_ = 3.23 ± 1.94 nm (Fig. 6*C*), which was almost the same as that for CCK1R/2R.

As a low endogenous level of CCK1R/2Rs may be expressed in CHO-GPR173 cells, the CCK2R-specific agonist CCK4 (Lin et al., 1989) and CCK1R-specific agonist A-71 623 (Lin et al., 1991) were applied to exclude false-positive signals. As expected, CHO-CCK2R and CHO-CCK1R cells responded to CCK4 and A-71 623, respectively, in a concentration-dependent manner, but neither could induce Ca^2+^ release in CHO-GPR173 cells (Fig. 6*D*,*E*). We then tried to knock down the expression of GPR173 by applying shRNA (Gpr173) in CHO-GPR173 cells (Fig. 6*F*). The Ca^2+^ signal was downregulated by >50% after the mRNA level of GPR173 was knocked down by >50% (Fig. 6*G*). GPR173, also known as SREB3, is highly evolutionarily conserved and predominantly expressed in the CNS (Matsumoto et al., 2005; Regard et al., 2008). The endogenous ligands and physiological functions of GPR173 have not properly and directly been determined in the brain. A previous study of the phoenixin (PNX)/GPR173 system illustrates that PNX is essential for regulating the mRNA of GPR173 in the pituitary (Treen et al., 2016). However, from our cell-based assays, the CCK ligand CCK8s binds to GPR173 with nm potency. In contrast, PNX did not directly trigger an immediate Ca^2+^ response (Fig. 6*H*) or β-arrestin recruitment (Vandevoorde et al., 2018). Thus, PNX may modulate GPR173 activity or expression through an unknown pathway that needs further investigation.

These results indicate that the CCK8s-induced Ca^2+^ response in CHO-GPR173 cells was mediated by GPR173, again supporting that GPR173 is a novel CCK receptor.

### GPR173 recruits β-arrestin after activation by CCK8s

CCK1R was reported to recruit β-arrestin1 (Ning et al., 2015). CCK2R was reported to recruit both β-arrestin1 and β-arrestin2 (Magnan et al., 2011, 2013). Of the four forms of arrestins (arrestin1, arrestin2, arrestin3, and arrestin4), arrestin2 and arrestin3, also named β-arrestin1 and β-arrestin2, respectively, are expressed in the brain (Gurevich and Gurevich, 2006; Latapy and Beaulieu, 2013).

In addition to the cell surface binding and Ca^2+^ imaging assays, we also applied a β-arrestin2 recruitment assay (PRESTO-Tango assay; Kroeze et al., 2015) to verify whether GPR173 is a CCK receptor. However, no β-arrestin2 recruitment was found for GPR173 after incubation with CCK8s (Fig. 6*L*). Consequently, we coexpressed β-arrestin1–conjugated TEV protease in the β-arrestin2 recruitment assay to detect whether GPR173 could recruit β-arrestins. We named this method the β-arrestin1/2 recruitment assay (Fig. 6*I*). We found that the β-arrestin1/2 recruitment signal for CCK1R was much higher than that in the β-arrestin2 assay (Fig. 6*J*), which is in line with previous reports that CCK1R could recruit β-arrestin1 (Ning et al., 2015). Meanwhile, the β-arrestin1/2 recruitment efficiency of CCK2R was far more than that in the β-arrestin2 recruitment assay (Fig. 6*K*), indicating that our modified β-arrestin recruitment system is reliable and more efficient. Next, we checked the dose-dependent response of CCK8s-induced β-arrestin recruitment for GPR173. We found that GPR173 responded to CCK8s in the β-arrestin1/2 recruitment assay (Fig. 6*L*).

In summary, CCK8s-induced β-arrestin recruitment further supports that GPR173 is likely a novel CCK receptor.

### GPR173 is necessary for HFLS-induced iLTP

**Figure 7.**
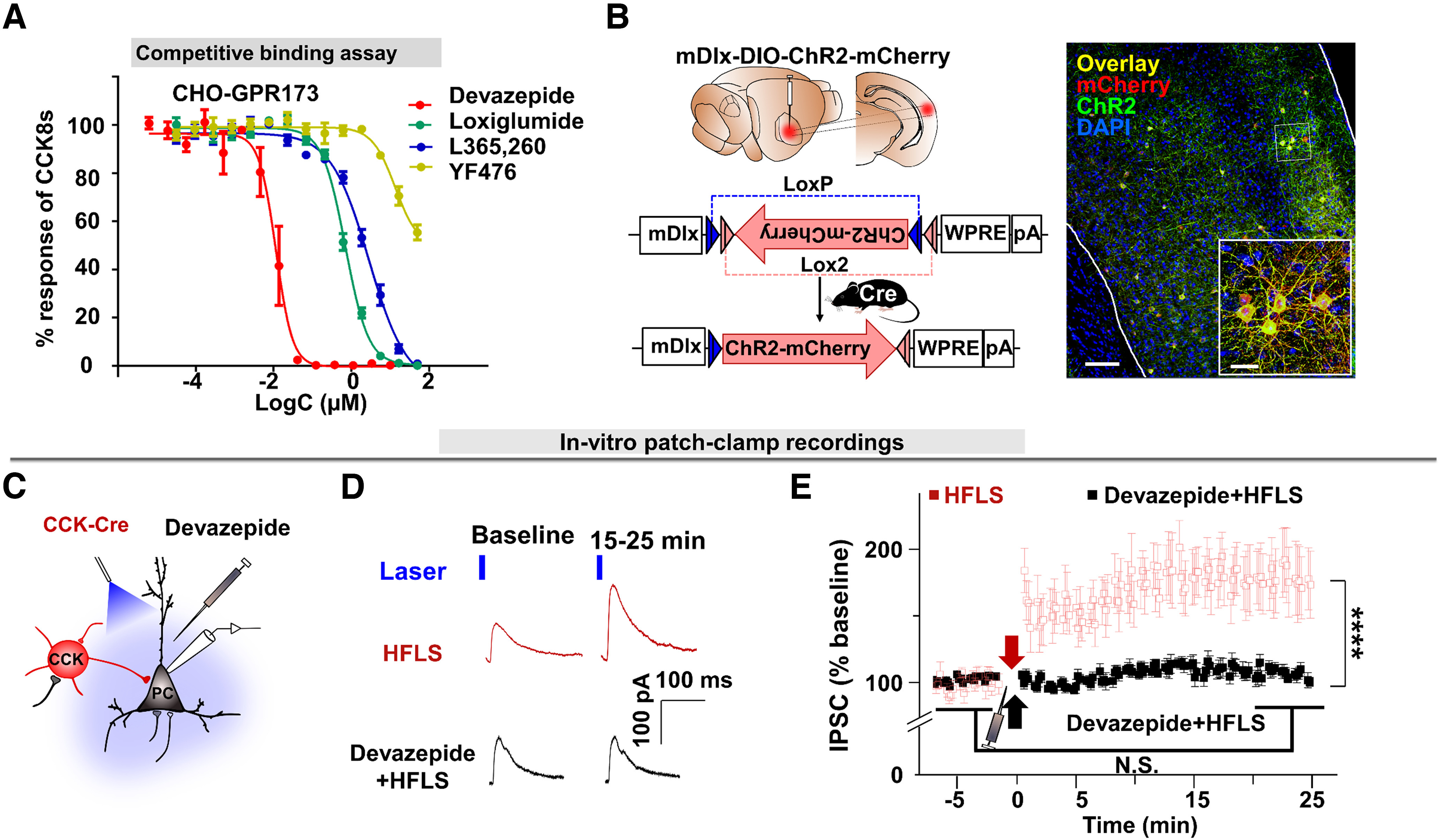
Devazepide as a GPR173 antagonist could block HFS-induced iLTP. ***A***, The dose-dependent response curve of CHO-GPR173 cells activated by 10 nm CCK8s after incubation with antagonists for 30 min (at least *n* = 3 independent experiments). ***B***, Injection of rAAV9-mDlx-DIO-ChR2-mCherry into CCK-Cre and PV-Cre mice. ***C***, Schematic drawing shows *in vitro* patch-clamp recording from a target PC neuron while delivering a laser pulse to activate infected CCK neurons (top). ***D***, Traces of IPSC responses to HFLS in brain slices from CCK-ires-Cre mice before and after HFLS (red) or devazepide + HFLS (black). ***E***, Time course of normalized IPSCs before (for 5 min) and after (for 25 min) HFLS in CCK-ires-Cre mice or after (for 25 min) devazepide plus HFLS in CCK-Cre mice (black, *n* = 9 neurons; one-way ANOVA with Tukey's *post hoc* test, N.S. not significant; two-way RM ANOVA with Tukey's *post hoc* test, ****p < 0.0001). Data are expressed as mean ± SEM.

Considering the homology between CCK1R/2R and GPR173, we hypothesized that some reported CCK1R/2R antagonists might act on GPR173 and block CCK8s-induced Ca^2+^ responses. Thus, we selected several well-known CCK1R/2R antagonists that might inhibit CCK8s-induced Ca^2+^ responses in GPR173-CHO cells, including CCK1R antagonists devazepide (Bauer et al., 2018) and loxiglumide (Beglinger et al., 2001), and CCK2R antagonists YF476 (Mohammed et al., 2019) and L365,260 (Wu et al., 2014). Among these CCK1R/2R antagonists, devazepide had the highest antagonistic action on GPR173 with an IC_50_ of 12.3 nm for CCK8s-induced Ca^2+^ responses (Fig. 7*A*). Then, we examined whether devazepide could block HFLS-induced potentiation of inhibition in brain slices from mDlx-DIO-ChR2-mCherry-injected CCK-Cre mice using a patch-clamp recording protocol (Fig. 7*B*). We selectively patched pyramidal neurons in the virus-injected area of the AC (Fig. 7*C*) and found that the application of devazepide (60 nm in bath solution) fully blocked the HFLS-induced potentiation of IPSCs (Fig. 7*D*; individual and averaged traces of IPSCs; Fig. 7*E*; group data, 100.18 ± 1.91% vs 103.24 ± 2.54%, baseline vs 20–25 min after devazepide infusion with HFLS, two-way ANOVA with Tukey HSD, ****p < 0.0001, *n* = 8), in contrast with HFLS without application of devazepide (HFLS vs devazepide plus HFLS, 183.70 ± 6.25%, *n* = 9, vs 103.24 ± 2.54%, *n* = 8, two-way RM ANOVA, *post hoc* with Tukey HSD, ****p < 0.0001). The Ca^2+^ and patch-clamp assay also suggest that devazepide is an antagonist for GPR173. Although devazepide antagonizes both CCK1R and GPR173, the blockade of iLTP occurred through its antagonism of GPR173, as iLTP was not shown to be mediated by CCK1R or CCK2R (Fig. 3).

In summary, this evidence further supports that GPR173, a novel CCK receptor, mediates the CCK signal from CCK-INs and induces iLTP in their synapses.

## Discussion

Because of the complexity of inhibitory neurons and connections, heterogeneous forms of inhibitory plasticity exist. Previous investigators proposed mechanisms for enhanced inhibitory control expressed at either the presynaptic or postsynaptic level. Different mechanisms of inhibitory plasticity at the presynaptic level that have been proposed include the release of more GABA transmitters (Caillard et al., 1999; Fiszman et al., 2005; Nugent et al., 2007), increased PV expression (Patz et al., 2004), and a GABAergic inhibition-induced decrease in glutamate release (Pan et al., 2009). iLTP is induced by postsynaptic activation of VGCCs (Kang et al., 1998; Caillard et al., 1999; Udakis et al., 2020). VGCCs provide a Ca^2+^ source for activating CaMKII (Udakis et al., 2020), which induces GABA_A_R phosphorylation and leads to iLTP (Petrini et al., 2014; He et al., 2015; Chiu et al., 2018). A study on awake mice identified a group of GABAergic neurons that burst or fire vigorously during rest and sharp-wave ripples and stay silent during locomotion and theta states (Szabo et al., 2022). CCK-INs are most active when the animal is at rest, whereas PV-INs remain silent (Dudok et al., 2021). However, most of these studies focus on inhibition-regulating behaviors depending on the hippocampus. More detailed mechanisms of iLTP from *in vivo* or long-term behavioral experiments remain elusive. Excitatory input arrives at the cell together with an inhibitory counterpart when an external stimulus activates the local cortex, which is normally recognized as the foundation of balanced cortical circuits (Galindo-Leon et al., 2009; Vogels et al., 2011; Alluri et al., 2016). We found that high-frequency activation of GABAergic neurons potentiated their long-term inhibition toward their target neurons and enhanced the general inhibition of the surrounding region, as reflected in the integrated neuronal responses to the forthcoming AS (Fig. 1). This iLTP in a the brain of a mature animal is critical for imbalanced excitation- and inhibition-induced brain disorders in adults (Vogels et al., 2011).

Considering the diverse electrophysiological, molecular, and structural features of GABAergic neurons, functional and behavioral correlates of this diversity are to be clarified accordingly. For example, PV-INs take leading control of the fast and powerful inhibitory effect on local neural activity, which makes them vital for the synchronization of spikes and synchronous gamma (30–80 Hz) oscillations (Kvitsiani et al., 2013; Chen et al., 2017; Jang et al., 2020). Stronger synchronization of PV-IN activity acts as a functional unit in the local medial prefrontal cortex, promoting goal-directed behavior (Kim et al., 2016). SOM-INs inhibit distal dendrites of excitatory neurons, causing them to act as key regulators of whisker movements in the somatosensory cortex (Yu et al., 2019) and the acquisition of motor skills in the motor cortex (Chen et al., 2015). Dendritic iLTP of SOM-IN synapses is driven by Ca^2+^ influx through NMDARs, whereas inputs from PV-INs and VIP-INs are unaffected (Chiu et al., 2018). These findings indicate the possibility of circuit-specific plasticity of GABAergic neurons.

In the visual cortex, high-frequency electrical stimulus trains (50 Hz) induce GABA_A_R-dependent LTP of inhibitory postsynaptic potentials of pyramidal neurons in developing rats (Komatsu, 1994). HFS of the subthalamic nucleus increases extracellular striatal GABA in hemiparkinsonian rats, which may benefit patients suffering from severe parkinsonism (Bruet et al., 2003). In the ventral tegmental area, HFS also potentiates IPSCs of GABAergic synapses, and this heterosynaptic LTP is NMDA receptor and GABA_A_R dependent (Nugent et al., 2007). In these previous studies, LTP was induced by electrical HFS, which activates all types of excitatory and inhibitory neurons and passing fibers and terminals. Here, we targeted only GABAergic neurons, specifically CCK-INs, and applied HFLS to investigate their postsynaptic connectivity with pyramidal cells using the whole-cell recording. We clearly demonstrate that iLTP of GABAergic output to pyramidal neurons was induced after HFLS of GABAergic neurons (Fig. 2). Our study focused on CCK-GABAergic output to pyramidal neurons. As CCK is colocalized in some VIP neurons (Gonchar, 2008), Lee et al. (2013) claim that some VIP-INs preferentially inhibit SOM-INs, increasing their activity during whisking and leading to disinhibitory control in the cortex by inhibiting SOM-INs or PV-INs (Pfeffer et al., 2013; Pi et al., 2013). Whether and how iLTP occurs among CCK-INs and other GABAergic neurons remains to be investigated.

CCK-GABAergic synapses modulate GABA release to the target PCs via presynaptic type 1 cannabinoid receptor (CB1R; Lee et al., 2010). The inability to potentiate inhibition to a forthcoming AS after HFLS of local GABAergic neurons in Vgat-Cre-CCK-cKO mice suggests that CCK is crucial for iLTP (Fig. 1), although this observation is because of less CCK-induced decreased GABA release via presynaptic GPR173 or CB1R, which requires further study. CCK-INs directly project to the soma of pyramidal cells (Fig. 5*G*; Kawaguchi and Kubota, 1998), supporting our observation that IPSCs (with a short latency of 4.53 ± 0.28 ms, *n* = 10 cells) of pyramidal cells induced by optogenetically activating CCK-INs is a directly induced response rather than a triggered response of crossing mutineuron nodes (Fig. 2). The significant potentiation of IPSCs caused by HFLS of CCK-INs and the induction of iLTP in the presence of exogenous CCK8s after LFLS of CCK-INs further validates the critical role of CCK in iLTP induction (Fig. 2). Moreover, the anxiolytic effect of CCK-INs in the ventral hippocampus of rats (Moghaddam et al., 2012) and the observation that CCK-IN activity inversely scales with pyramidal cell activity (Dudok et al., 2021) suggests that CCK-INs also play a functional role in the hippocampus. How CCK-INs switch inhibitory plasticity in other brain areas and how they interact with other GABAergic neurons in inhibitory plasticity remain to be explored.

CCK1R and CCK2R were discovered in the brain several decades ago (Van Dijk et al., 1984; Hill et al., 1987), which was followed by investigations of their gene expression (Honda et al., 1993) and associated behaviors and disorders (Reubi et al., 1997; Horinouchi et al., 2004; Cayrol et al., 2006). Later research mainly focused on the signaling, biological activities, and structure of these two characterized CCK receptors (Ning et al., 2015; Zeng et al., 2020; Ding et al., 2022), with less attention paid to identifying new receptors. The induction of iLTP in CCK1R/2R-KO mice after HFLS of CCK-INs (Fig. 3) suggests that an unknown CCK receptor (i.e., CCK3R) mediates this synaptic plasticity. Because of receptor expression levels, the probing efficiency of ligands, and the readout of autoradiographic experiments, the possibility remains that unknown receptors with low expression and selective binding potency with specific isoforms of CCK were missed during our characterization. Based on our *in vivo* and *in vitro* recording experiments, we believe that new CCK receptors are awaiting clarification, which will significantly challenge our current understanding. Using different bioinformatics algorithms, we narrowed the range of CCK receptor candidates. We demonstrated that GPR173 is a novel CCK receptor by histology and three independent cell-based assays (Figs. 4–6). CCK8s-induced iLTP in cortical slice recordings (Fig. 2), Ca^2+^ mobilization, and recruitment of β-arrestin in the cell line assay (Fig. 6) possibly occur through binding and activation of GPR173. GPR173 receptors are expressed specifically in excitatory neurons rather than inhibitory cells and are localized in CCK-GABAergic synapses in the AC (Fig. 5*E*,*F*). A GPR173 antagonist, devazepide, entirely blocked HFLS-induced iLTP, confirming the role of GPR173 in iLTP induction (Fig. 7).

Combining the current understanding with our new finding of CCK3R, we propose several possible mechanisms for iLTP induction. The activation of CCK3R by CCK release leads to Ca^2+^ signaling, possibly through the opening of Ca^2+^ channels in postsynaptic neurons or the release of Ca^2+^ from the endoplasmic reticulum. iLTP may also require an increase of gephyrin and postsynaptic GABA_A_Rs and CaMKII activation, as in previous studies (Petrini et al., 2014; He et al., 2015; Chiu et al., 2018; Udakis et al., 2020). Our cell-based assays indicate CCK application triggers a Ca^2+^ signal. Ca^2+^ signaling potentially enables GPR173-mediated iLTP via the following mechanisms: (1) by increasing GABA_A_R insertion in the postsynaptic surface membrane through promoting exocytosis (Petrini et al., 2014), (2) by increasing mobilization of GABA_A_Rs in the postsynaptic cell, or (3) by stabilizing GABA_A_R or VGCC gating properties. Similar to glutamatergic synapses, GABAergic synapses undergo homosynaptic and heterosynaptic plasticity (Ravasenga et al., 2022). In the current study, we could not exclude the possibility that CCK-IN–induced iLTP is restricted to the CCK postsynaptic area without affecting neighboring PV-IN synapses or other synapses. Whether this GPR173-mediated iLTP is homosynaptic or heterosynaptic remains to be investigated in the future.

In summary, we demonstrated that HFLS of local GABAergic neurons induced iLTP, which was CCK dependent. We report a novel CCK receptor, GPR173, localized in CCK-GABAergic synapses that mediates CCK-mediated iLTP. The involvement of CCK and its new receptor CCK3R in iLTP indicates their crucial roles in enhancing the inhibitory level in the neocortex and possibly other regions, allowing maintenance of the excitation and inhibition balance of the brain. As CCK3R mediates the potentiation of iLTP rather than directly inhibits neuronal activity, it may provide a better target for treating brain disorders related to excitation/inhibition balance, such as epilepsy, schizophrenia, and depression.
